# Down-regulation of peptidylarginine deiminase type 1 in reconstructed human epidermis disturbs nucleophagy in the granular layer and affects barrier function

**DOI:** 10.1038/s41420-023-01509-8

**Published:** 2023-06-29

**Authors:** Adebayo Candide Alioli, Julie Briot, Carole Pons, Hang Yang, Marie Gairin, Dominique Goudounèche, Laura Cau, Michel Simon, Marie-Claire Méchin

**Affiliations:** 1 Toulouse Institute for Infectious and Inflammatory Diseases (Infinity), University of Toulouse, CNRS, INSERM, University Paul Sabatier, Toulouse, France; 2 Centre de Microscopie Électronique Appliquée à la Biologie (CMEAB), University of Toulouse, Medical Faculty of Toulouse, Toulouse, France; 3 Present Address: University of Lyon, INSERM UMR1033, Lyon, France; 4 Present Address: Capgemini, Issy les Moulineaux, France; 5 Present Address: Pierre Fabre Dermo-Cosmétique, Muret, France; 6 Present Address: Silab, Saint-Viance, France

**Keywords:** Post-translational modifications, Nuclear organization

## Abstract

Deimination is a post-translational modification catalyzed by a family of enzymes named peptidylarginine deiminases (PADs). PADs transform arginine residues of protein substrates into citrulline. Deimination has been associated with numerous physiological and pathological processes. In human skin, three PADs are expressed (PAD1-3). While PAD3 is important for hair shape formation, the role of PAD1 is less clear. To decipher the main role(s) of PAD1 in epidermal differentiation, its expression was down-regulated using lentivirus-mediated shRNA interference in primary keratinocytes and in three-dimensional reconstructed human epidermis (RHE). Compared to normal RHEs, down-regulation of PAD1 caused a drastic reduction in deiminated proteins. Whereas proliferation of keratinocytes was not affected, their differentiation was disturbed at molecular, cellular and functional levels. The number of corneocyte layers was significantly reduced, expression of filaggrin and cornified cell envelope components, such as loricrin and transglutaminases, was down-regulated, epidermal permeability increased and trans-epidermal-electric resistance diminished drastically. Keratohyalin granule density decreased and nucleophagy in the granular layer was disturbed. These results demonstrate that PAD1 is the main regulator of protein deimination in RHE. Its deficiency alters epidermal homeostasis, affecting the differentiation of keratinocytes, especially the cornification process, a special kind of programmed cell death.

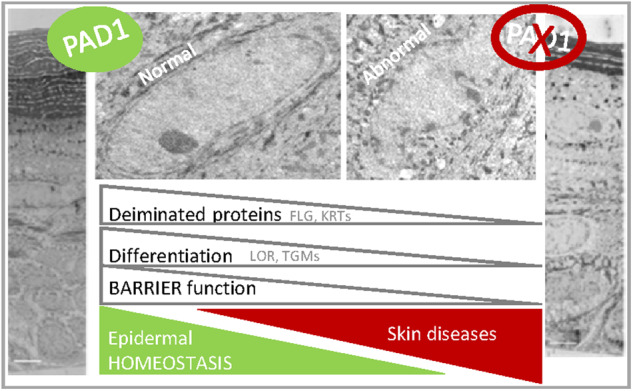

## Introduction

Deimination, or citrullination, is a calcium-dependent post-translational modification of arginine to citrulline residues catalyzed by peptidylarginine deiminases (PADs). Deimination alters the charge of targeted proteins thereby causing changes in their conformation and in interactions with partners. In mammals, five conserved PAD isotypes exist [PAD1-4 and PAD6], encoded by distinct and clustered *PADI* genes [1, 2]. PADs display different tissue expression and are involved in many physiological and pathological processes [3–7].

Three PADs (PAD1-3) are expressed in the human skin [8]. Recently, mutations of human PAD3 have been described in hair and scalp diseases, affecting hair follicle shape [9] and causing a particular alopecia with perifollicular inflammation [10]. However, the specific role of each PAD isotype in the epidermis remains unknown. PAD1 is present in the cytoplasm of keratinocytes and in the matrix of corneocytes, from the basal to the most superficial cornified layer [8, 11, 12]. The ubiquitous PAD2 is detected in all suprabasal layers of living keratinocytes [8, 13]. PAD3 is mainly expressed in granular keratinocytes and in the deepest corneocyte layers [8, 14].

For years, the characterized epidermal targets of PADs were filaggrin, keratins K1 and K10, trichohyalin, filaggrin-2, and hornerin [12, 15–17]. Using a proteomic approach, we identified new substrates such as the keratinocyte proline rich protein, a cornified envelop component that is important for the epidermal barrier [4, 18]. In three-dimensional reconstructed human epidermis (RHE), PAD inhibition by the pan inhibitor Cl-amidine slows down cornification, the last step of keratinocyte differentiation at the granular to cornified cell transition and disturbs the associated autophagy process [19]. PAD1 is suspected of regulating the full degradation of filaggrin, a key protein for the skin barrier function, and hence the production of the filaggrin degradation-related natural moisturizing factor. Indeed, we demonstrated that reducing external relative humidity increases expression of PAD1 (with no impact on the expression of other PADs) and drives human filaggrin breakdown by increasing its deimination rate [20]. Therefore, deimination may play an active role in protecting human skin and in *Stratum corneum* hydration in physiological conditions. PAD1 has also recently been shown to be less immunodetected in lesional skin of psoriatic patients and to be down-regulated by IL-22 [21].

The aim of the present study was to decipher the specific role of PAD1 in human epidermis. PAD1 expression was down-regulated using RNA interference in human primary keratinocytes used to produce RHEs. The effects of PAD1 down-regulation on RHE morphology, on epidermal barrier function, and on the expression of several differentiation markers were analyzed. An impact of PAD1 down-regulation on granular keratinocyte nucleophagy was highlighted (see graphical abstract).

## Results

### RHE produced at 50% relative humidity

RHEs were produced in an incubator with relative humidity adjusted to 50% (Supplemental Material and Fig. S1 online). At day 10 (D10), compared to day 4 (D4), RHEs displayed all the characteristics of normal human epidermis, with the four constitutive layers (cornified, granular, spinous, and basal) (Fig. S1A). A marked increase in PAD1 expression and in the deimination rate was observed between D10 and D4 with ratios of about 7 and 14, respectively (Table S1 and Fig. S1B–D), confirming the already known induction of PAD1 at the mRNA and protein levels during keratinocyte differentiation in vitro [19, 21] and in vivo [11]. As expected, profilaggrin and filaggrin were largely detected at mRNA and protein levels (Fig. S1B–D). In this study, RHEs were produced at 50% relative humidity and harvested at D10.

### *PADI*1 down-regulation in RHEs almost turns off protein deimination

**Fig. 1:**
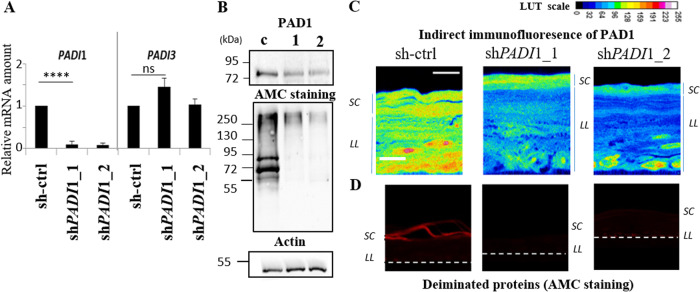
Efficiency and specificity of PAD1 down-regulation. **A** RT-qPCR of RHEs transduced by sh-ctrl, sh*PADI*1_1, and sh*PADI*1_2. Relative mRNA steady states of *PADI*1 and *PADI*3 are reported, other *PADI* mRNA were undetectable (Cycle threshold, Ct > 35). **B**–**D** Expression of PAD1, deiminated proteins, and actin. **B** Western blot analysis of total extract RHEs transduced by sh-ctrl, sh*PADI*1_1, and sh*PADI*1_2. **C** PAD1 immunofluorescence of RHE sections after transduction by sh-ctrl, sh*PADI*1_1, and sh*PADI*1_2. Representation using a LUT scale reported to highlight the down-regulation of PAD1 in sh*PADI*1_1 and shPADI1_2 compared to sh-ctrl RHEs. **D** sh-ctrl, sh*PADI*1_1, and sh*PADI*1_2 RHEs were analyzed by indirect immunofluorescence to localize deiminated proteins (AMC staining) in situ. The line at the bottom represents the polycarbonate filter. *SC* *Stratum corneum*, *LL* living cell layers. Scale bars*,* 10 µm.

Down-regulation of PAD1 expression was performed using lentivirus-mediated shRNA interference in normal human primary keratinocytes obtained from five different donors, and the transduced keratinocytes were used to produce RHEs. Two shRNAs targeting *PADI*1 were validated (sh*PADI*1_1, and sh*PADI*1_2) as well as a control shRNA (sh-ctrl), the sequences of which are reported in Table S2. *PADI*1 down-regulation were probed by RT-qPCR, Western blotting, and using an indirect immunofluorescence assay. Effective and specific down-regulation of PAD1 expression was obtained at the mRNA level with both sh*PADI*1 compared to sh-ctrl (89.50 ± 11.36% for sh*PADI*1_1 and 90.00 ± 1.00% for sh*PADI*1_2) while the amount of *PADI*3 was maintained, and *PADI*2, 4 and 6 mRNA remained undetectable (Figs. 1A, 3D and data not shown). Therefore, no compensatory variations were observed in other *PADI*s. At the protein level, the detection of PAD1 in sh*PADI*1 RHE extracts was reduced to 58.40 ± 6.50% of controls (Fig. 1B, top panel). Consistently, PAD1 was weakly immunodetected in sections of sh*PADI*1_1 and sh*PADI*1_2 compared to sh-ctrl RHEs (Fig. 1C). Importantly, deiminated proteins were barely detected in both sh*PADI*1 RHEs, either by Western blotting (Fig. 1B, middle panel) or by indirect immunofluorescence (Fig. 1D). Only one band (>250 kDa) was weakly detected in the sh*PADI*1 RHE extracts compared to strong detection of numerous deiminated proteins in controls. The global rate of deimination in sh*PADI*1 decreased significantly (~85%) (Fig. 1B and Fig. S2A, B), and deiminated keratins in particular, almost disappeared (Fig. 1B and Fig. S2C, D).

### PAD1 deficiency in RHEs alters cornification and keratohyalin granules

**Fig. 2:**
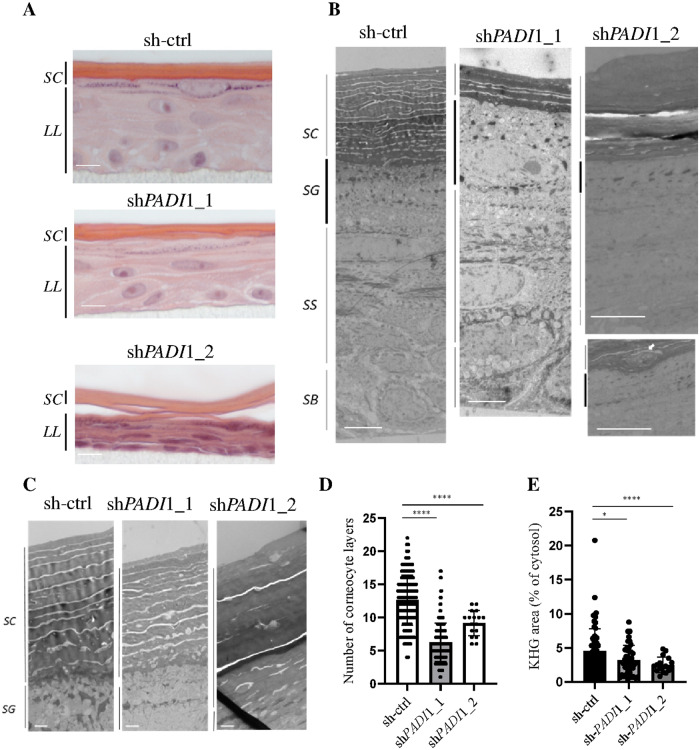
Tissue morphology and ultrastructural aspects of PAD1 down-regulated RHEs. **A** Hematoxylin and eosin staining of sh-ctrl, sh*PADI*1_1, and sh*PADI*1_2 RHEs. **B** Left to right: full vertical TEM sections of sh-ctrl, sh*PADI*1_1, and sh*PADI*1_2 RHEs. **B** Right bottom, white arrow: parakeratotic nucleus in sh*PADI*1 RHEs. **C** *Stratum corneum* of sh-ctrl, sh*PADI*1_1, and sh*PADI*1_2 RHEs. **D** Number of corneocyte layers of RHEs (mean ± SD) for sh-ctrl and sh*PADI*1_1, *n* = 5; sh*PADI*1_2, *n* = 2. **E** KHG area of RHEs. (sh-ctrl and sh*PADI*1_1, *n* = 5; sh*PADI*1_2, *n* = 2). **A**, **B** Black lines: *LL living cell layers,* *SC* *Stratum corneum*, *SG* *S. granulosum (bold line)*, *SS* *S. spinosum*, *SB* *S. basale*. Scale bars = 10 µm (**A**), 5 µm (**B**), 1 µm (**C**).

RHE morphology was visualized by hematoxylin/eosin coloration and by transmission-electronic microscopy (TEM) (Fig. 2). At the histological level, when PAD1 was down-regulated, the thickness of the epidermis, in particular that of the cornified layer, seemed to decrease, and keratohyalin granule (KHG) staining was less visible (Fig. 2A). TEM analysis confirmed that the structure of sh*PADI*1 RHEs was normal (Fig. 2B) compared to controls, but had a thinner *Stratum corneum* with a significant decrease in the number of corneocyte layers (mean number = 12.64 ± 3.81 for the sh-ctrl RHEs versus 6.27 ± 2.87 for sh*PADI*1_1 and 9.18 ± 1.91 for sh*PADI*1_2 RHEs; *p* < 0.0001) (Fig. 2C, D). Parakeratotic nuclei were sometimes observed in both sh*PADI*1 but not in sh-ctrl RHEs (Fig. 2B, bottom right panel). In addition, the relative KHG surface area relative to the granular keratinocyte cytoplasmic area was significantly reduced [4.53 ± 3.28% for the sh-ctrl versus 3.21 ± 2.14% for sh*PADI*1_1 (*p* = 0.0117) and 2.48 ± 1.15% for sh*PADI*1_2 RHEs (*p* < 0.0001)] (Fig. 2C, E). These observations suggest that keratinocyte homeostasis, and consequently the proliferation/differentiation balance, may be impaired when PAD1 is down-regulated.

### PAD1 deficiency does not disturb keratinocyte proliferation

**Fig. 3:**
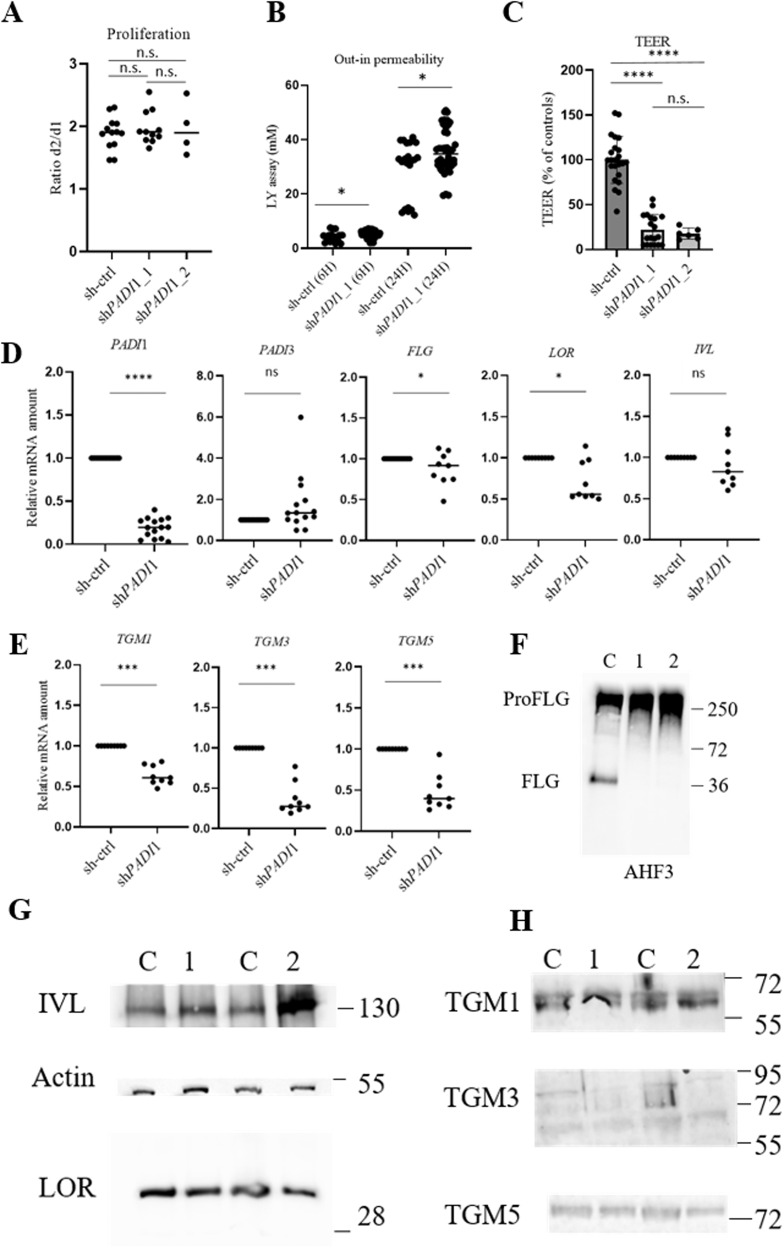
Effect of PAD1 down-regulation on proliferation, differentiation, and barrier function. **A** Proliferation capacity of transduced NHEK by sh-ctrl (*n* = 11), sh*PADI*1_1 (*n* = 11), and sh*PADI*1_2 (*n* = 4). **B** LY permeability of sh-ctrl and sh*PADI*1_1 RHEs (at 6 h, *p = 0.0317; at 24 h, *p = 0.0163; *n* = 7, RHEs; 2 banks). **C** Relative percentage of TEER for sh-ctrl, sh*PADI*1_1, and sh*PADI*1_2 RHEs (*n* = 6). **D**, **E** RT-qPCR analysis for **D** *PADI*1, *PADI*3, (pro)filaggrin (*FLG*), loricrin (*LOR*), involucrin (*IVL*) and for **E** transglutaminase (*TGM*) 1, 3 and 5 in sh-ctrl and sh*PADI*1 RHEs. Data were pooled for statistical analysis: sh*PADI*1_1 *n* = 7 to 13 and sh*PADI*1_2 *n* = 2 from 5 banks. **F**–**H** Immunodetections of total protein extracts of sh-ctrl (C), sh*PADI*1_1 (1), and sh*PADI*1_2 (2) RHEs as indicated. Ladder sizes indicated on the right (kDa).

To evaluate the potential defect in keratinocyte proliferation, we cultured post-transduced keratinocytes in a monolayer for two days, and calculated the number of cells at D1 and D2 (Fig. 3A). The ratio was similar whatever the condition (1.89 ± 0.26 for the sh-ctrl, 1.98 ± 0.27 for sh*PADI*1_1, *p* = 0.4324 and 1.97 ± 0.43 for sh*PADI*1_2, *p* = 0.6524). These data demonstrated that PAD1 deficiency did not alter keratinocyte proliferation.

### PAD1 deficiency affects the epidermal barrier function of RHEs

The effect of PAD1 down-regulation on *Stratum corneum* functionality was assessed using several complementary assays. First, inside-out permeability was investigated by measuring trans-epidermal water loss (TEWL). No significant differences were found between sh-ctrl and sh*PADI*1_1 RHEs (Fig. S3A). In addition, the pH at the surface of RHEs was the same (equal to ~7.0) (Fig. S3B). Next, the outside-in permeability was investigated using the lucifer yellow (LY) assay (Fig. 3B). The amount of LY in the culture medium 6 and 24 h after its application on the top of RHEs was slightly but significantly increased by the down-regulation of PAD1 compared in the control condition; e.g., after 24 h, the mean concentrations were 29.11 ± 10.45 mM for sh-ctrl versus 35.74 ± 8.31 mM for sh*PADI*1_1, *p* = 0.0163. Finally, mean trans-epidermal-electric resistance (TEER) values were drastically decreased in both sh*PADI*1 (2205 ± 2090 ohm/cm² for sh*PADI*1_1 and 1656 ± 992 ohm/cm² for sh*PADI*1_2) compared to sh-ctrl RHEs (9657 ± 6977 ohm/cm², *p* < 0.0001, Fig. 3C). This result could be explained by modulations of mRNA and protein amounts of claudin-1 and occludin (Fig. S4A and S4D). Taken together, these results show that the barrier function is impaired in PAD1-deficient RHEs.

### PAD1 deficiency reduces the expression of transglutaminases

To further decipher the effect of PAD1 deficiency on cell differentiation and corneocyte formation at the molecular level, mRNA of other markers of keratinocyte differentiation were analyzed by RT-qPCR in RHEs produced with keratinocytes from up to five donors.

In sh*PADI*1 RHEs, the relative profilaggrin and loricrin mRNA levels were slightly decreased (0.8 and 0.5 fold, respectively) whereas no significant variations were observed in the levels of involucrin, corneodesmosin, desmocollin-1 and desmoglein-1 (Fig. 3D and Fig. S4B and S4D). A significant decrease in transcripts was measured for transglutaminases 1, 3, and 5 (0.5, 0.2, and 0.4 fold, respectively) (Fig. 3E). Some of the corresponding proteins were immunodetected by Western blotting (Fig. 3F–H). In PAD1 deficient extracts, a clear reduction in the detection of filaggrin monomers was observed (29.31 ± 24.28% relative to controls) while profilaggrin remained similar to controls (105.44 ± 40.64%) (Fig. 3F). The levels of loricrin and involucrin detected were also equivalent (Fig. 3G). In agreement with the RT-qPCR data, the amount of the native form (higher band, ~72 kDa) of tranglutaminases 3 and 5 detected decreased in the two sh*PADI*1 (Fig. 3H). Furthermore and as expected, the in situ transglutaminase 1 activity showed a peripheral pattern in the late differentiated keratinocytes but with no significant modulations (Fig. S5).

### PAD1 deficiency affects the shape of nuclei in the granular keratinocytes

**Fig. 4:**
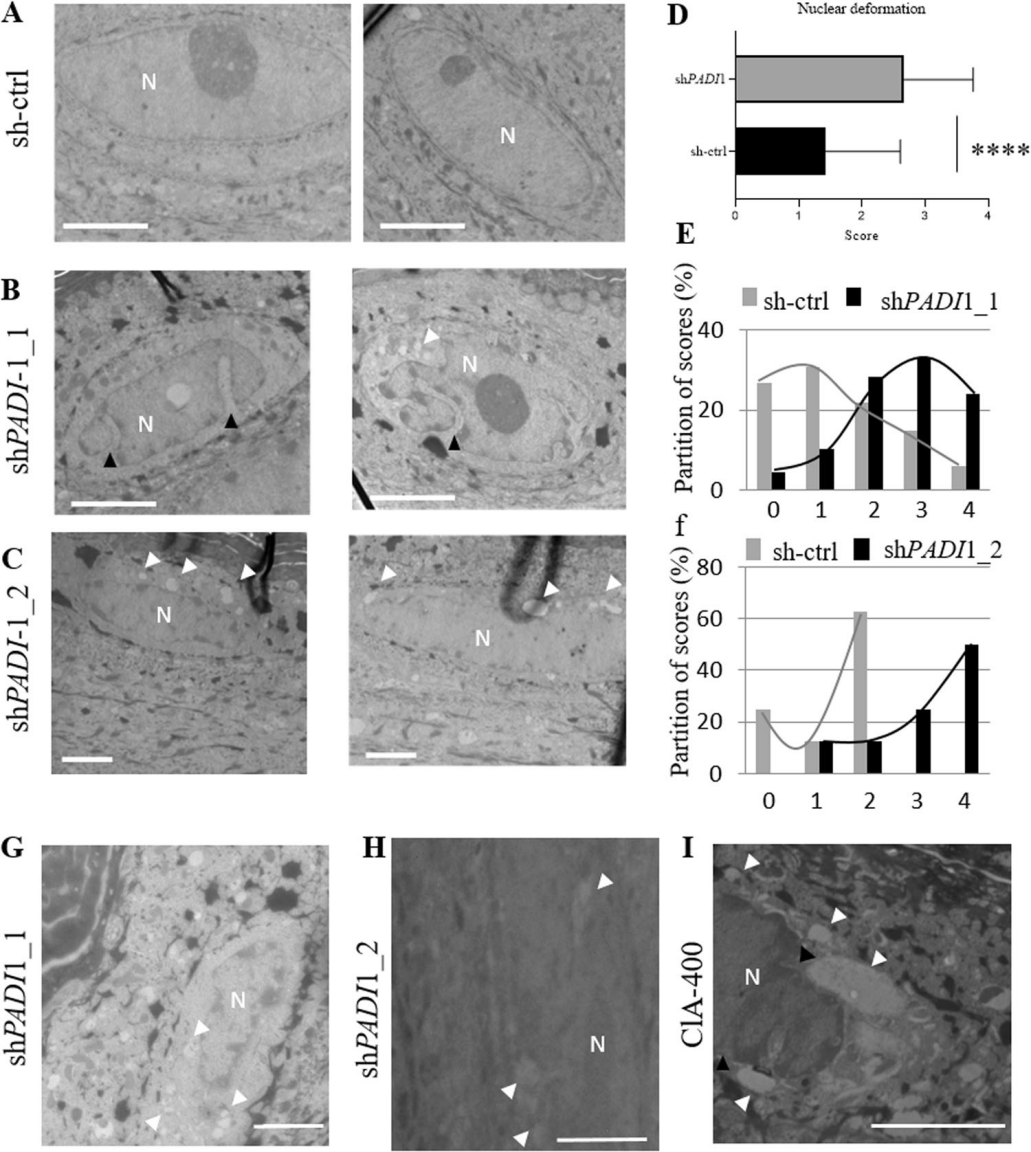
Effect of PAD1 down-regulation on nuclear shape. **A**–**C** Representative illustrations of nuclear shape for sh-ctrl, sh*PADI*1_1, and sh*PADI*1_2 RHEs, respectively. **B** Deep nuclear deformations (or invaginations) indicated by black arrows. **D** Nuclear deformation scoring of sh-ctrl (109 nucleus) and sh*PADI*1 (96 nucleus) RHEs, NHEK banks, *n* = 5; 7 independent experiments, *n* = 6 for sh-ctrl, sh*PADI*1_1 and 1 for sh*PADI*1_2. **E** Distribution of nuclear deformation scores for sh-ctrl (gray) and sh*PADI*1_1 (black) RHEs. **F** Distribution of nuclear deformation scores for sh-ctrl (gray) and sh*PADI*1_2 (black) RHEs. **G**, **H** Representative perinuclear vesicles (small circular white area) indicated by white arrows. Also in (**B**) and (**C**). **I** Representative images of perinuclear vesicles (white arrows) and deep invaginations (black arrows) of the nuclear envelope in RHE treated with 400 µM Cl-amidine (ClA-400). Scale bars, 2 µm. N, nucleus.

TEM images highlighted changes in the nuclear shape in the granular layer of sh*PADI*1 RHEs compared with controls (Fig. 4A–C). As illustrated in Fig. S6, these deformations were scored using images corresponding to seven independent RHE productions (7 RHEs for sh-ctrl, 6 for sh*PADI*1_1, and 1 for sh_*PADI*1_2). The score for sh*PADI* RHEs was almost doubled, with a mean ± SD equal to 1.42 ± 1.18 for sh-ctrl versus 2.66 ± 1.10 for sh*PADI*1 RHEs (*p* < 0.0001) (Fig. 4D). The distribution of scores for sh*PADI*1_1 and sh*PADI*1_2 RHEs were similar (Fig. 4E, F). Furthermore, deep invaginations in the nuclei were observed, as illustrated in Fig. S6E, F (black arrow), for 29.17% of sh-ctrl versus 68.75% for sh*PADI*1 RHEs (*p* = 4.6561e-7). These data demonstrated the major impact of PAD1 deficiency on nuclei at the granular to cornified cell transition. In addition, vesicles around nuclei of granular keratinocytes were notable and were scored (Fig. 4G, H). A significant increase in vesicles was observed in PAD1 deficient RHEs compared to controls [scores equal to 0.75 ± 0.69 for sh-ctrl versus 1.12 ± 0.79 for sh*PADI*1 RHEs (*p* = 0.00219)]. These observations led us to analyze the expression of four autophagic key markers at protein and mRNA levels. PAD1 deficiency did not appear to have a major impact on the expression of these markers (Fig. S4C, D).

In a previous work, we observed cytosolic and perinuclear vesicles in the differentiated keratinocytes of RHEs treated with Cl-amidine, an irreversible inhibitor of PADs [19]. We therefore performed new TEM observations of the Cl-amidine-treated RHEs, focusing on nuclei. Nuclear deformations were significantly increased, with scores of 1.93 ± 1.30 for controls versus 3.09 ± 0.99 for RHEs treated with 400 µM, and 2.62 ± 1.23 for RHEs treated with 800 µM of Cl-amidine (Fig. S7A). The distribution of the scores for nuclear deformation and for perinuclear vesicles were also modulated by the PAD inhibitor (Fig. 4I and Fig. S7A-D). Furthermore, after 24 h of treatment with Cl-amidine, the proliferation capacity of keratinocytes was similar to control cells (Fig. S8).

Taken together, these results support a major role for PAD1 and deimination in the nucleophagy process during the late differentiation of keratinocytes in human epidermis, at the transition between granular to cornified layers.

## Discussion

In this study, tridimensional RHEs produced in 50% relative humidity were first characterized. A fully differentiated and stratified epidermis was obtained ten days after exposure to an air-liquid interface, with characteristics similar to the normal human interfollicular epidermis. In this model, the filaggrin monomer was increasingly detected during the course of the epidermis reconstruction process. The expression of PAD1 and 3 was confirmed, and as expected, deiminated proteins were detected in the cornified layer. These data are in line with previous results showing that relative humidity of 30–50% promotes epidermal differentiation [20, 22].

Using this model, we showed that down-regulation of PAD1 with two distinct shRNAs caused almost total suppression in the overall protein deimination. Only one deiminated protein with a high molecular mass (> 250 kDa) remained, which may correspond to the known PAD3 target trichohyalin [9, 14, 16]. This demonstrates that PAD1 is the main isotype involved in epidermis protein deimination, in full agreement with the recent hypothesis of its major role in the regulation of filaggrin breakdown [20].

Further, this work showed that PAD1 deficiency caused a dysregulation of keratinocyte differentiation, with a thinner cornified layer and a decrease of KHG. The functional properties of sh*PADI*1 RHEs were disturbed with a small increase in outside-in permeability (LY assay) and, a huge collapse of the TEER, but no significant impact on inside out permeability (TEWL values) and surface pH. This deleterious phenotype could be partially explained by the lower number of corneocyte layers and by tight junction defects, as shown by the modulation of claudin-1 and occludin expression. In sh*PADI*1 RHEs, the amount of filaggrin monomers was lower. Transglutaminases 3 and 5 were drastically down-regulated at both the mRNA and protein levels. Although no clear impacts were detected for involucrin and loricrin, two major components of the cornified envelope [23], we suspect that less transglutaminases could affect the protein or lipid composition and properties of the envelopes. This is in line with the impact on RHEs of treatments with the PAD inhibitor, Cl-amidine [19] (Table 1). Conversely, when PAD1 expression and the protein deimination rate were induced in RHEs produced under low relative humidity, the expression of transglutaminase 5 and the number of corneocyte layers increased [20] (Table 1).

**Table 1:** Characteristics of RHEs with impaired PAD1 activity.

	Dry atmosphere	Cl-A inhibitor^a^	sh*PADI*1
Characteristics	Cau et al., 2017	Cau et al., 2019	This work
Deimination/citrullination	Hypercitrullination	Hypocitrullination	Hypocitrullination
Deiminated proteins/PAD activity	Increase (~5-8x)	Decrease (~50%)	Decrease (~ 85%)
mRNA/*PADI*1	Increase (~12x)	n.d.	Decrease (~ 90%)
mRNA/other *PADI*s	No compensatory effect^b^	n.d.	No compensatory effect^b^
PAD1 protein	Increase (~4x)	n.d.	Decrease (~ 40%)
RHE morphology	Thicker *SC*	Similar	Thiner *SC*
Keratinocyte proliferation	Unchanged	Unchanged	Unchanged
Transitional cells	Not observed	Increased number	Not observed
Keratohyalin granules	Increase (~1.6x)	n.d.	Decrease (~ 0.7-0.5x)
Profilaggrin	Tendency to increase	Decrease (~0.8x)	Decrease (~ 0.8x)
Filaggrin (monomer)	Decrease	Decrease (~ 0.8x)	Decrease (~ 0.3x)
Deiminated filaggrin	Increase	Decrease	Decrease
Other differentiation markers	IVL (~1.6x), LOR (~0.6x)	IVL unchanged n.d.	IVL unchanged, LOR (~ 0.5x)
Claudin-1 (tight junction protein)	n.d.	n.d.	Increase (~ 1.5x)
Desmosomal proteins	CDSN (1.6x)	n.d.	CDSN, DSG1, DSC1 unchanged
Transglutaminases	TGM5 increase (~2x)	TGM5 decrease (~0.04x)	TGM1,3,5 decrease (~0.2-0.5x)
Number of corneocyte layers	Increase (~2x)	Decrease	Decrease (~0.5–0.7x)
TEWL (inside-out)	Decrease	n.d.	Unchanged
Surface pH	Decrease	n.d.	Unchanged
LY permeability (outside-in)	Unchanged	n.d.	Increase
TEER	n.d.	n.d.	Decrease (~80%)
Autophagic markers (LC3B, p62, etc.).	n.d.	Increase	Unchanged
Autophagic process	n.d.	Disturbed	Unchanged
Nucleophagic process	n.d.	Disturbed	Disturbed
Parakeratosis	Not observed	n.d.	Observed
Perinuclear vesicles	Not observed	Increase	Increase
Nuclear deformation	Not observed	Increase	Increase
Phenotypes	Inverse	Similar	

^a^Chloro-amidine (Cl-A) is an inhibitor that irreversibly binds to the cysteine of the active site of all PADs.

^b^*PADI*3 ~, others undetectable; n.d., not determined.

Finally, down-regulation of PAD1 in RHEs caused major changes in nuclear shape in the *Stratum granulosum*, with deep nuclear invaginations, and the occurrence of perinuclear vesicles. Nuclear removal is a well-known characteristic of granular keratinocyte to corneocyte transition. Autophagy and particularly nucleophagy has been recently proposed as involved mechanisms [24, 25]. In our study, no impact on autophagic mRNA markers was observed, suggesting that autophagy was not induced by down-regulation of PAD1 expression. We observed similar morphological modifications when PADs were inhibited with Cl-amidine (Table 1). However, in the latter case, expression of autophagic markers increased [19]. One possible explanation is that down-regulation by RNA interference acts during the full period of RHE production with a marked decrease in the deimination rate, whereas Cl-amidine treatment was performed only during the last 48 h leading to a reduction of only ~50% in PAD activity. Nevertheless in both cases, nucleophagy is clearly disturbed when PAD expression and/or activity decreases in RHEs.

Nucleophagy during cornification is beginning to be deciphered at the cellular and molecular levels [24–27]. This requires Akt serine threonine kinase 1-dependent phosphorylation of lamins A/C inducing their subsequent dispersal and degradation, actin filament remodeling, and DNA degrading enzymes, namely DNase1L2 and DNase2. The present work is the first demonstration of the involvement of PAD1 and deimination, as a new post-translational modification, in this process. Lamin C carboxy terminus has been reported to be deiminated by PAD4 in nuclear fragmentation during apoptosis of several cell types [5, 28]. A 70 kDa perinuclear protein has also been proposed to be deiminated during in vitro apoptotic events in rat differentiated keratinocytes following calcium ionophore induction [29]. Deimination of similar nucleo-proteins and keratins (K1 and K10) by PAD1 could be involved in nuclear shape deformations in late keratinocyte differentiation steps.

In conclusion, this work shows that knockdown of *PADI*1 in RHEs almost completely inhibits protein deimination. This leads to the collapse of TEER, a reduced number of corneocyte layers and KHG area, altered keratinocyte differentiation (especially transglutaminases, filaggrin and tight junction proteins) and impaired nucleophagic processes, highlighting the major role of PAD1 in epidermal homeostasis at the granular to cornified cell transition. Consequently, it seems to be important to take post-translational modifications such as deimination into account to better decipher keratinocyte physiology and skin diseases.

## Materials and methods

### shRNA lentiviral particles

PAD1 down-regulation in keratinocytes was performed with two distinct shRNA targeting *PADI*1 (Table S2) and using the technical procedures previously reported [30].

### Primary normal human keratinocyte culture, transduction, and production of RHEs

Primary normal human epidermal keratinocytes (NHEK banks) were produced, with the approval of the French Minister of Research, as previously described [20]. For knockdown experiments, keratinocyte suspensions were infected by lentivirus particles containing either sh*PADI*1*_*1, sh*PADI*1_2, or the sh*-*ctrl at a multiplicity of infection of five, at 37 °C, 5% CO_2_. RHEs were produced at the air-liquid interface with controlled 50% relative humidity, and harvested at day 10 as previously described [19, 20, 31] and as detailed in Supplemental Material and at Fig. S1.

### Proliferation capacity

During puromycin selection, after medium refreshment, ten images of adherent transfected keratinocytes (magnification ×4, EVOS digital inverted microscope, AMG) were randomly taken. Using the software imageJ (NIH, NY), the numbers of adherent keratinocytes were evaluated to calculate ratios between the cell numbers at days 2 and 1.

### Western blotting

Total epidermal proteins were extracted in Laemmli sample buffer and immunodetected with antibodies listed in Table S3, as previously described [20].

### Tissue staining and immunostaining

RHEs were fixed in formaldehyde and embedded in paraffin. Five-micron thick sections were observed after a hematoxylin-eosin staining or immunodetected with the listed antibodies, as previously described [20].

### Functional measurements

TEWL and pH at the surface of RHEs were measured as previously described [20] and see Supplemental Material. LY (Sigma Aldrich, FR) permeability was quantified after 6 and 24 h of incubation as previously described [20] and Supplemental Material. Trans-epidermal-electric resistance (TEER) was evaluated using a Millicell ERS-2 apparatus, on 19 RHEs for sh*PADI1*_1 (produced from 4 banks), on 6 RHEs for sh*PADI1*_2 (1 bank), and compared to 23 RHEs for sh-ctrl (4 banks).

### Reverse-transcription and quantitative real-time PCR

Total RNA extraction, reverse transcription, and real-time PCR were performed as previously reported [32]. RNA quality was confirmed for RIN > 8.5, on an Agilent 2100 Bioanalyzer, using a “RNAs 6000 Nano” kit according to the manufacturer’s instructions (Agilent, US), as previously described [19]. Quantitative PCR amplification was performed with the 7300 Real-Time PCR System (Applied Biosystems, Foster City, CA) using the “Sybr quantitative PCR SuperMix W/ROX” (Invitrogen Life Technologies, Fr.). The specific primer pairs are listed in Table S4.

### Transmission electron microscopy: *Stratum corneum* cell layers and nuclear scoring

RHEs were processed on an HT7700 electron microscope (Hitachi, Tokyo, Japan), as previously described [19, 20]. The number of *Stratum corneum* cell (corneocyte) layers was quantified in at least three independent locations, and compared in sh-ctrl versus sh*PADI*1*_*1 (*n* = 6 banks) or sh*PADI*1_2 (*n* = 2 banks). Nuclear deformation was scored on a 0 to 4 scale, using images at ×2500 magnification (Fig. S6A–E). The absence or presence of at least one deep invagination of the nuclear envelope was scored 0 or 1, respectively (Fig. S6E, F). The perinuclear vesicles were scored 0, 1, or 2 according to their absence, abundance, and size.

### Statistical analysis

A Shapiro-Wilk normality test was performed, and differences were evaluated using a parametric Student’s *t*-test, Welch’s *t*-test, or non-parametric Wilcoxon’s test using the GraphPad Prism (V 9.3.1). The statistical significance threshold was set at 0.05 (*p < 0.05; **p < 0.01; ***p < 0.001; ****p < 0.0001).

## Supplementary information

Supplementary information final version

Original Data File

## Data Availability

The datasets generated during and/or analyzed during the current study are available from the corresponding author on reasonable request.
