# Supplementary material for: Down-regulation of peptidylarginine deiminase type 1 in reconstructed human epidermis disturbs nucleophagy in the granular layer and affects barrier function
Source: Cell Death Discov. 2023 Jun 29;9:198. doi: 10.1038/s41420-023-01509-8 (PMC10310762; doi:10.1038/s41420-023-01509-8)
Supplement: Supplementary file 1 — Supplementary information final version [file 41420_2023_1509_MOESM1_ESM.docx]

**Down-regulation of human peptidylarginine deiminase type 1 disturbs nucleophagy in the granular layer and epidermal barrier function**

Adebayo Candide Alioli, Julie Briot, Carole Pons, Hang Yang, Marie Gairin, Dominique Goudounèche, Laura Cau, Michel Simon and Marie-Claire Méchin

**Supplemental Material**

**Supplemental material and methods**

**shRNA lentiviral particles**

For PAD1 down-regulation in keratinocytes, two distinct MISSION pLKO.1-puro vector–based lentiviral particles (Sigma-Aldrich, Fr.) were validated. They contained a puromycin resistance gene and a PAD1-directed shRNA insert under the control of the human U6 promoter. The targeted PAD1 mRNA sequences correspond to the 3’UTR region (sh*PADI1*_1) at nucleotide position 2328–2348 on the mRNA sequence (NCBI Refseq NM_013358.3) and to the coding region (sh*PADI1*_2) at position 1774-1795. A shRNA (sc-002-LV particle) that does not target any known human gene (sh-ctrl) was used as control as previously reported [30]. For more details and the exact sequences of the shRNAs, see Table S2.

**Primary normal human keratinocyte culture, transduction and RHE production**

The primary normal human keratinocytes used for this study were produced from skin after abdominal dermolipectomy of five different healthy subjects (40.4±10.4 years old), obtained from Genoskin (Toulouse, FR) with a legal approval of the French Minister of Research. Normal human epidermal keratinocyte libraries (NHEK bank) were amplified and stored as previously described [20]. For shRNA knockdown experiments, keratinocytes were infected in suspension with lentivirus particles containing either a sh*PADI*1 (1 or 2) or the sh-ctrl at a multiplicity of infection of five in the presence of 8 µg/ml of protamine sulfate (Sigma-Aldrich). After 24 hours of incubation, transduced keratinocytes were selected in refreshed medium containing 2 µg/ml of puromycin (Sigma-Aldrich). When transduced keratinocytes reached 60–70% of confluence, they were harvested to produce RHEs as described previously, except that relative humidity in the cell incubator (INCO incubator; Memmert GmbH, Schwabach, Ge) was adjusted to 50% [19, 20, 31]. To evaluate this *in vitro* 3D model for the study of PADs and deimination, RHEs were harvested at day four (D4), day seven (D7) and day ten (D10) after the beginning of culture at the air–liquid interface.

**N/TERT-1 keratinocyte cell line : culture, Cl-amidine treatments and proliferation capacity**

The N/TERT-1, a human keratinocyte cell line [s1] was cultured for 24 hours with Dermalife medium as usual, at 37°C and 5% CO_2_, before to be treated without (control condition) or with 100 µM Cl-amidine (treated condition) for further 24 hours. To evaluate the proliferation capacity of keratinocytes, five images of cells in each well (at least n=8 for each) were acquired at random before the treatment (Day 1) and 24 hours latter (Day 2). The cell numbers were numerated for each image with the “multi-pointed tool” of the imageJ software, and mean ± SD of ratios (day 2 / day1) was evaluated for each condition.

To validate the Cl-amidine treatment, two autophagic markers (Sestrin 2 and LC3B) were analysed by western blotting as previously reported [19]. Quantification of detections was normalized to actin.

**Transepidermal water loss (TEWL) assay**

TEWL was measured with a Tewameter (TEWAMETER TM300, Courage & Khazaka, Cologne, Germany) according to the manufacturer's instructions, as previously described [20].

**Lucifer yellow (LY) permeability assay**

Five hundred µl of 1 mM Lucifer Yellow (Sigma-Aldrich) were added to the RHEs at day 10. After incubation at 37 °C for 6 or 24 h, the concentration of dye in the culture medium was measured fluorometrically using a VarioskanFlash (Thermo Fisher Scientific, Waltham, MA) with excitation and emission at 428 and 540 nm, respectively.

**pH measurement**

The surface pH of the RHEs was measured using the flat HI-99181a skin pH meter (HANNA instruments, Woonsocket, RI), after topic application of 2 µL of ultra-pure water to RHEs.

**Trans-epidermal-electric resistance (TEER)**

Using several RHEs produced from independent NHEK banks, TEER was measured in a petri dish cover using a Millicell ERS-2 as previously described, with 500 µL of 1X phosphate buffered saline at room temperature (Thermo Fisher Scientific) on top of each RHE and 5 mL outside [31]. TEER is expressed in ohm.cm².

***In situ* transglutaminase activity assay**

*In situ* transglutaminase activity (mainly transglutaminase 1 assay) was evaluated on RHE cryo-sections and compared between sh-ctrl and sh*PADI*1_1 on three RHE productions with independent NHEK banks. Positive transglutaminase cell layers and mean grey values of the red fluorescent labeling were evaluated using image-J, as previously described [19-20].

**Supplemental results**

**RHE produced at 50% relative humidity**

The general morphology of the reconstructed tissues was analyzed after hematoxylin-eosin staining and compared with the epidermis of the normal human abdominal skin used to prepare the primary normal human keratinocytes (Fig. S1A). The RHE harvested at D4 had no *Stratum corneum*. At D10, as expected, the RHE displayed all the characteristics of a normal epidermis, with the four different cellular layers: cornified, granular, spinous and basal layers. PADs and profilaggrin expression was then analyzed at the mRNA level (Fig. S1B). Between D4 and D10, the level of *PADI*1 mRNA increased significantly (7.8 fold), whereas the level of *PADI*3 mRNA did not vary and *PADI*2, 4 and 6 transcripts were not detected. Profilaggrin mRNA increased significantly (5.0 fold) from D4 to D10 (Supplementary data Fig. S1B). Protein deimination and the expression of profilaggrin (> 400 kDa) and filaggrin (37 kDa) were then analyzed by Western blotting. Total proteins were extracted in Laemmli sample buffer, and immunodetected with the AMC antibody specific for deiminated proteins, and with AHF3, a monoclonal antibody directed to human filaggrin (Fig. S1C). Deiminated proteins (AMC detection) were slightly detectable at D4, were easily detectable at D7 but mainly at D10 when the *Stratum corneum* was completely formed. Profilaggrin and filaggrin detection increased from D4 to D10 in agreement with the real time PCR analysis of *FLG*. To confirm these data, immuno-histochemical analysis were performed using the same antibodies on RHE and normal skin sections (Supplemental Material Fig. S1D). Deiminated proteins (AMC staining), not easily detectable at D4, were strongly detected in the *Stratum corneum* of the RHEs at D7 and at D10 as observed in the epidermis of normal human skin (Supplementary data Fig. S1D, top panels). AHF3 stained the upper part of the epidermis, and the intensity of staining increased from D4 to D10. At D10, the *Stratum granulosum* and *corneum* were heavily labeled as usually observed in the epidermis of normal human skin (Fig. S1D, bottom panels). In view of these results, the RHEs produced in a relative humidity adjusted to 50% were harvested at D10.

**Supplemental Tables**

**Table S1.** Global rate of deimination detection (AMC) in RHEs at days 4, 7 and 10

| Ratio of mean grey values | | |
| --- | --- | --- |
| Day 10 / Day 4 13.67 |  |  |
| Day 7 / Day 4 6.67 |  |  |
| Day 10 / Day 7 2.05 | | |

**Table S2**. Sequences of shRNAs and references of lentiviral particles

|  | Lentivirus # | Sequences | |  |
| --- | --- | --- | --- | --- |
| sh*PADI*1_1 | **SHCLNV-NM_013358 TRCN0000434710** | | 5’-CCGGAGAGGCTCTAGATCAACAATGCTCGAGCATTGTT  GATCTAGA GCCTCTTTTTTTG -3’ | |
| sh*PADI*1_2 | **SHCLNV-NM_013358 TRCN0000051116** | | 5’-CCGGGAGAGTGACATCGTGGACATTCTCGAGTTGGTG  CTCTTCATC TTGTTGTTTTT -3’ | |
| Sh-ctrl | **SHC016V**  **(non target)** | | 5’- CCGGCAACAAGATGAAGAGCACCAACTCGA  GTTGGTGCTCTTCATC TTGTTGTTTTT -3’ | |
|  |  | |  | |

**Table S3.** Primary antibodies

| Antigen | Antibody | Reference | Concentration/Dilution | |
| --- | --- | --- | --- | --- |
|  |  |  | **WB** | **IIF** |
| Claudin-1 | pAb15098 | [30] | 1 :500 | - |
| Corneodesmosin | clone G36-19 | [s2] | 0.5 µg/ml | - |
| Filaggrin | clone AHF3 | [s3] | 0.5 µg/ml | 0.5 µg/ml |
| Involucrin | clone SY5 | Sigma-Aldrich, Fr | 1:1000 | - |
| Loricrin | pAb AF62 | Covance, Princeton, NJ | 1:10000 | - |
| Deiminated proteins | AMC* | [15] | 1:5000 | 1:2000 |
| LC3B | 2775 | [19] | 1 :1000 | - |
| Peptidylarginine deiminase 1 | anti-PAD1 | [11]  [8] |  | 1:50 |
| Transglutaminase 1 | pAb A018 | Zedira, Darmstadt, Germany | 1:200 | - |
| Transglutaminase 3 | pAb A015 | Zedira | 1:200 | - |
| Transglutaminase 5 | pAb A008 | Zedira | 1:500 | - |

***** When anti-modified citrulline (AMC) antibodies were used, citrullyl residues were chemically modified before the saturation step by incubation at 37 °C in 0.0125% FeCl_3_, 2.3M H_2_SO_4_, 1.5M H_3_PO_4_, 0.25% diacetyl monoxime and 0.125% antipyrine for 1 h for Western blotting and for 3 h for indirect immunofluorescence, as previously described [15].

**Table S4.** List of primer pairs used in RT-qPCR

| ***Gene name*** | 5’-3’ sequence | Ref. |
| --- | --- | --- |
| ***ATF4*** | CAGCAAGGAGGATGCCTTCT  TCCTTCAAATCCATTTTCTCCAA | [19] |
| ***ATG5*** | GCAGATGGACAGTTGCACACA  TTTCCCCATCTTCAGGATCAA | [19] |
| ***CDSN*** | ACTGCTGCTGGCTGGTCT  AGAGCTTCTGGCACTGGAAA | [30] |
| ***CLDN1*** | TGGCATGAAGTGTATGAAGTGCTT  CCCCAATGACAGCCATCCT | [30] |
| ***DSC1*** | CATGGGTGGTCAGCCTTTCGGT  TCCTGATCCTGTACCTTCATTCGCA | [30] |
| ***DSG1*** | GAAGGCAGAAACGTGAATGGA  TTTTGGCGATTGGGTTCCT | [30] |
| ***FLG*** | GCAAGGTCAAGTCCAGGAGAA  CCCTCGGTTTCCACTGTCTC | [32] |
| ***IVL*** | GCCAGGTCCAAGACATTCAAC  GGGTGGTTATTTATGTTTGGGTGG | [32] |
| ***LC3B*** | ACCATGCCGTCGGAGAAG  ATCGTTCTATTATCACCGGGATTTT | [19] |
| ***LOR*** | CGAAGGAGTTGGAGGTGTTT  ACTGGGGTTGGGAGGTAGTT | [19] |
| ***OCLN*** | CCTTCACCCCCATCTGACTA  GCAGGTGCTCTTTTTGAAGG | [30] |
| ***PADI1*** | AGAGTGACATCGTGGACATTC  GCTCGTGGTAGGACAAGTAGTC | [32] |
| ***PADI2*** | GGTGGGATGAGCAGCAAGCGAATC  GAACAGAGCGGGCAGGTCAATGATG | [32] |
| ***PADI3*** | GCAGAGTGTGACATCATTGACATCC  GACCGCACCTTCTCCTCCAG | [32] |
| ***PADI4*** | CCACACGGGGCAAACTGTC  CAGCAGGGAGATGGTGAGGG | [32] |
| ***PADI6*** | CGTGGAGAAGTGCATTCACCTGAAC  GCCTCGCAAAGGACCTCTTGGG | [32] |
| ***TJP1*** | TGAGGCAGCTCACATAATGC  GGTCTCTGCTGGCTTGTTTC | [30] |
| ***TGM1*** | CCCCCGCAATGAGATCTACA  ATCCTCATGGTCCACGTACACA | [20] |
| ***TGM3*** | GGAAGGACTCTGCCACAATGTC  TGTCTGACTTCAGGTACTTCTCATACTG | [20] |
| ***TGM5*** | CGGAGCAGGTTGAGGACTGT  GAGGACTCCAAGGAAGACTTTCTG | [20] |
| ***SESN2*** | ACAAGTGTTGTGGCCTTCCTGAAC  ATGGGTGAATGGCAAGTAGGAGGT | [19] |
| ***YWHAZ***** | ACTTTTGGTACATTGTGGCTTCAA  CCGCCAGGACAAACCAGTAT | [32] |

**, All the amplicons were checked by Sanger sequencing.*

***, normalizer housekeeping gene.*

**Supplemental figures and legends**

**
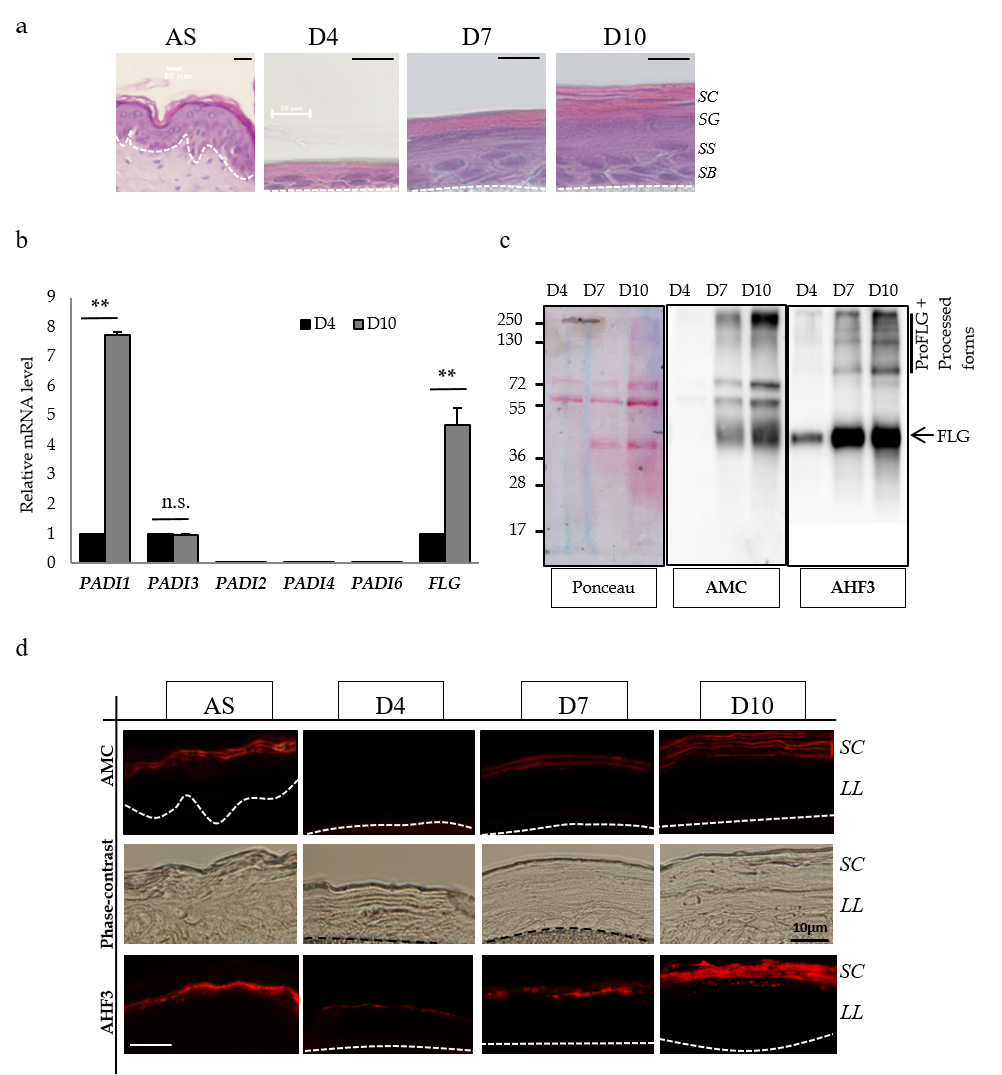
**

**Supplemental Fig. S1**. **Expression of PADs and detection of deiminated proteins in RHEs produced at 50% relative humidity. (A)** Sections of paraffin-embedded normal abdominal skin (AS) and RHEs harvested after 4, 7 and 10 days of culture at the air-liquid interface (D4, D7 and D10) were stained with hematoxylin and eosin. The *Stratum corneum* (sc), *Stratum granulosum* (sg), *Stratum spinosum* (ss) and *Stratum basale* (sb) are shown. The polycarbonate filter membrane for RHE (bottom limit) and dermo-epidermal junctions for normal skin are indicated by the white dashed line. (**B)** Total RNAs were isolated from RHEs harvested at D4 and D10, and analyzed by RT-qPCR using the amount of *YWHAZ* mRNA as a reference for normalization. The steady-state mRNA amounts of *PADI*1-6 and profilaggrin (*FLG*) are expressed relative to the amount at D4. Wilcoxon tests were performed to compare the mean mRNA levels at D10 and D4; the threshold for statistical significance was set at 0.05 (** *p* < 0.01; ns, non-significant). (**C)** Total proteins extracted from RHEs harvested at D4, D7 and D10 were separated by SDS-PAGE, transferred to nitrocellulose membrane, stained with Ponceau red and immunodetected with an antibody directed to deiminated proteins (anti-modified citrulline, AMC) and with the AHF3 monoclonal antibody specific for profilaggrin (ProFLG), FLG and processed intermediate forms, as indicated. Molecular mass markers are indicated in kDa on the left. (**D)** Sections of paraffin-embedded AS and RHEs at D4, D7 and D10 were analyzed by immunofluorescence staining, with AMC (top panels), AHF3 (bottom panels) and were observed with a phase contrast microscope (middle panels). The *Stratum corneum* (*SC*) and the leaving layers (*LL*) are shown. Bars = 10 µm.


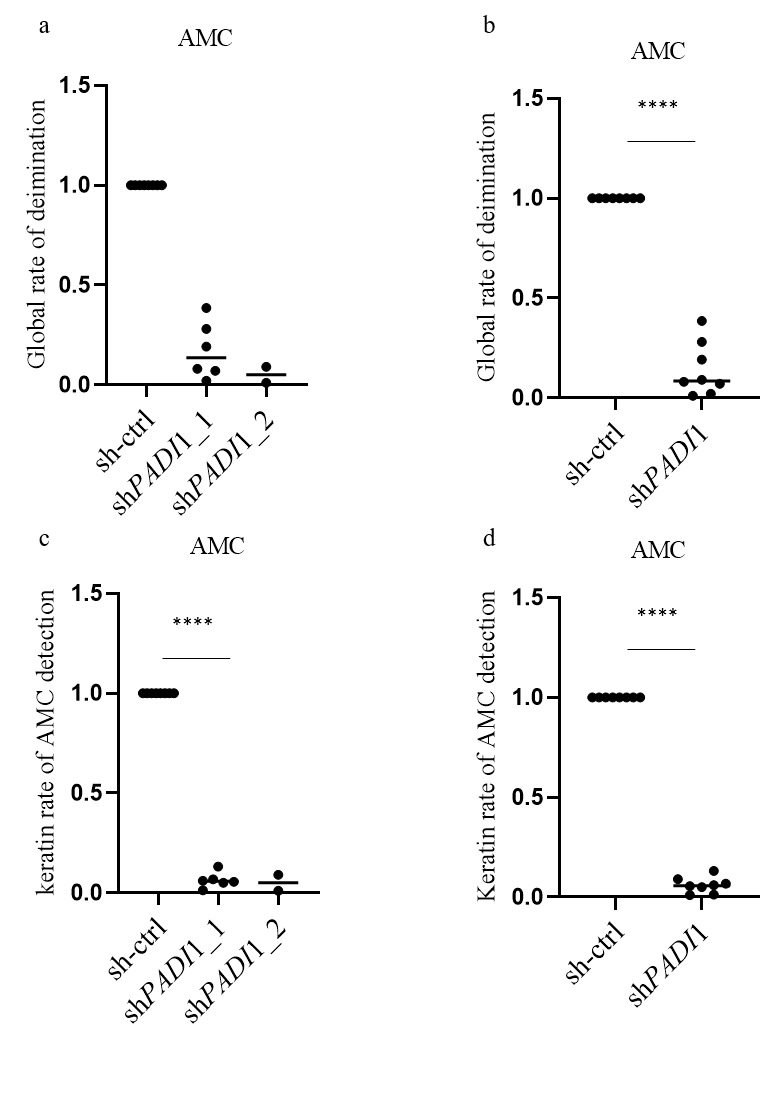


**Supplemental Fig. S2**. **Relative rate of deiminated proteins. (A and B)** Global relative rate of deiminated proteins in sh-ctrl, sh*PADI*1_1, sh*PADI*1_2 RHEs (**A**) and sh*PADI*1 (_1 and _2 cumulated) (**B**). (**C and D)** Relative rate of keratin deamination in sh-ctrl, sh*PADI*1_1, sh*PADI*1_2 RHEs (**C**) and shPADI1 (**D**). Each point corresponds to an individual RHE. ****, *p* < 0.0001.


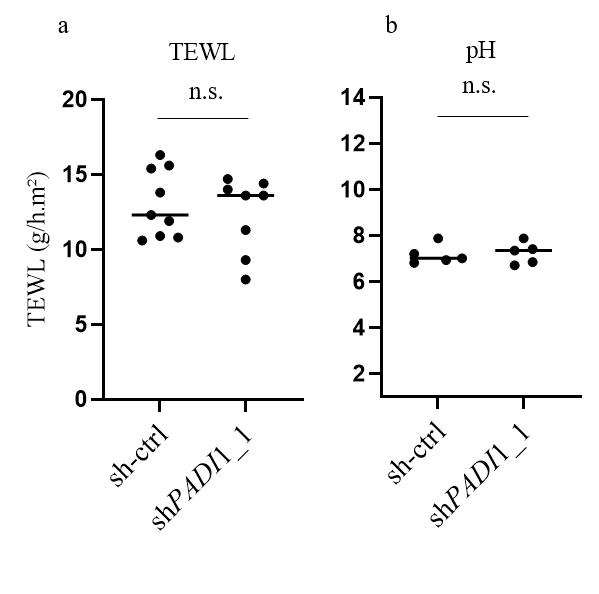


**Supplemental Fig. S3**. **Effect of PAD1 down-regulation on RHE permeability and surface pH.** (**A)** sh-ctrl and sh*PADI*1_1 RHE permeability was assessed. (**B)** The surface pH of sh-ctrl and sh*PADI*1_1 RHEs was also measured. For each condition, the tests were performed with five to nine RHEs obtained from two independent NHEK banks. ns, non-significant.

**
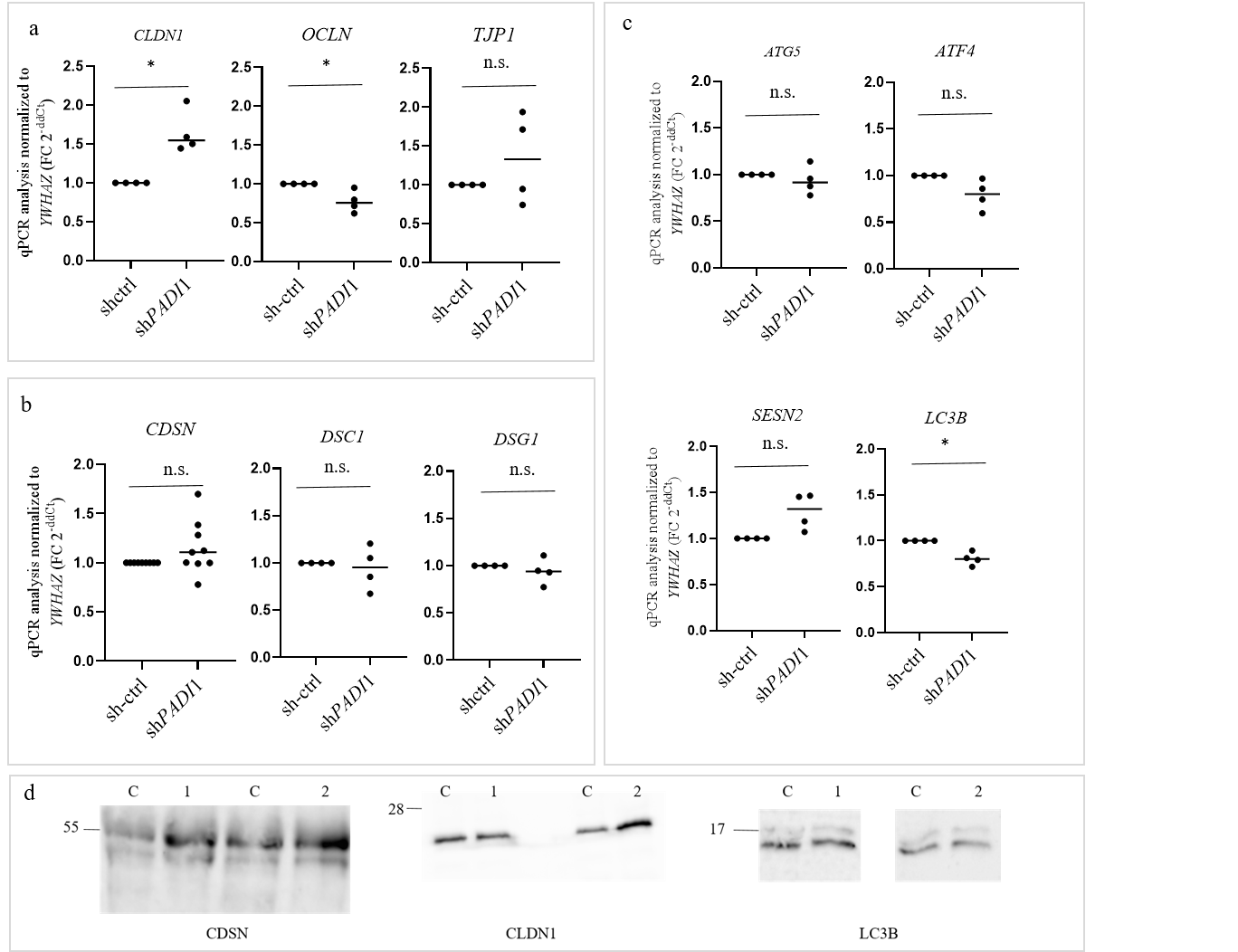
**

**Supplemental Fig. S4**. **Effect of PAD1 down-regulation on tight junction, desmosome and autophagic markers at mRNA and protein levels.** **(A-D)** sh-ctrl and sh*PADI*1 RHEs were analyzed by RT-qPCR (**A-C**) and Western blotting (**D**). Genes encoding (**A**) tight junction proteins: claudin-1 *(CLDN1)*, occludin *(OCLN)* and tight junction protein-1 (*TJP1*), (**B)** desmosomal proteins: corneodesmosin (*CDSN*), desmocollin-1 (*DSC1*) and desmoglein-1 (*DSG1*), (**C)** autophagic proteins: autophagic gene 5 (*ATG5*), autophagic transcription factor 4 (*ATF4*), sestrin-2 (*SESN2*) and microtubule-associated protein 1 light chain 3 beta (MAP1LC3B or LC3B). Individual data from sh*PADI*1_1 RHEs (n = 2 to 7) and sh*PADI*1_2 RHEs (n = 2) were pooled for statistical analysis using Graph Pad software 9.3.1 (ns, non-significant; *, *p* < 0.05). (**D)** Immunodetection of total proteins from sh-ctrl (C), sh*PADI*1_1 (1) and sh*PADI*1_2 (2) RHEs. On the left, molecular mass of markers in kDa. All the RT-qPCR and Western blotting analyses were performed as previously described (Méchin et *al*., 2010; Pendaries et *al*., 2014; Cau et *al*., 2017; Cau et *al*., 2019).

**
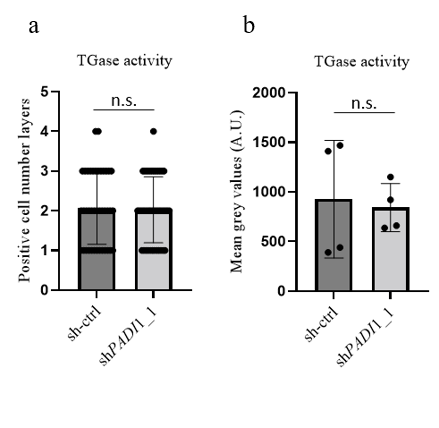
**

**Supplemental Fig. S5**. ***In situ* transglutaminase activity** for sh-ctrl and sh*PADI*1_1 RHEs (n=3 banks). **(A**) Number of positive cell layers. (**B**) Mean grey values of fluorescence intensity.

**
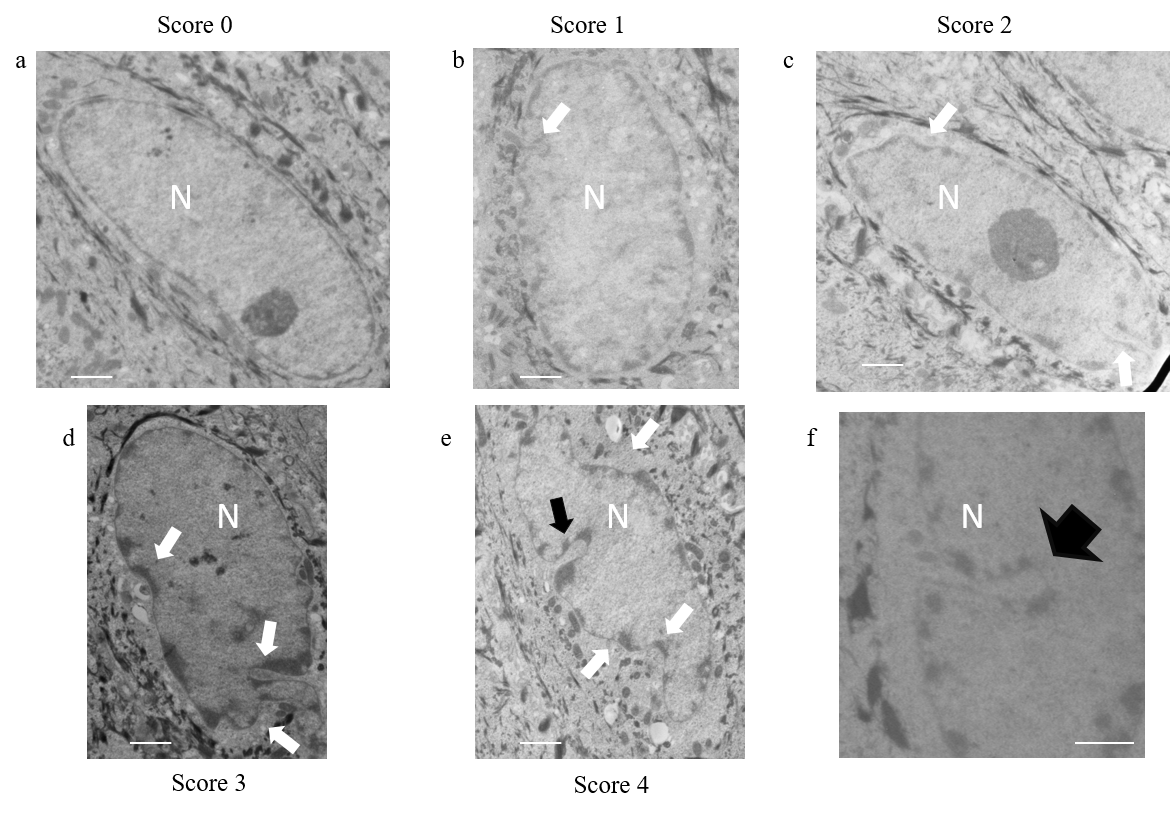
**

**Supplemental Fig. S6. Scoring of nuclear shape modifications induced by PAD1 down-regulation.** TEM image 2500x magnification. (**A-E)** Illustrations of nuclear deformations with the corresponding score from 0 to 4. (**F)** Enlargement of a deep invagination of the nuclear membrane. The presence of a deep invagination was scored 1, its absence was scored 0. Bars = 1 µm. Seventy-two to 222 nuclei were observed. White arrows point to nuclear deformations. Black arrows point to deep invaginations. N, nucleus.

**
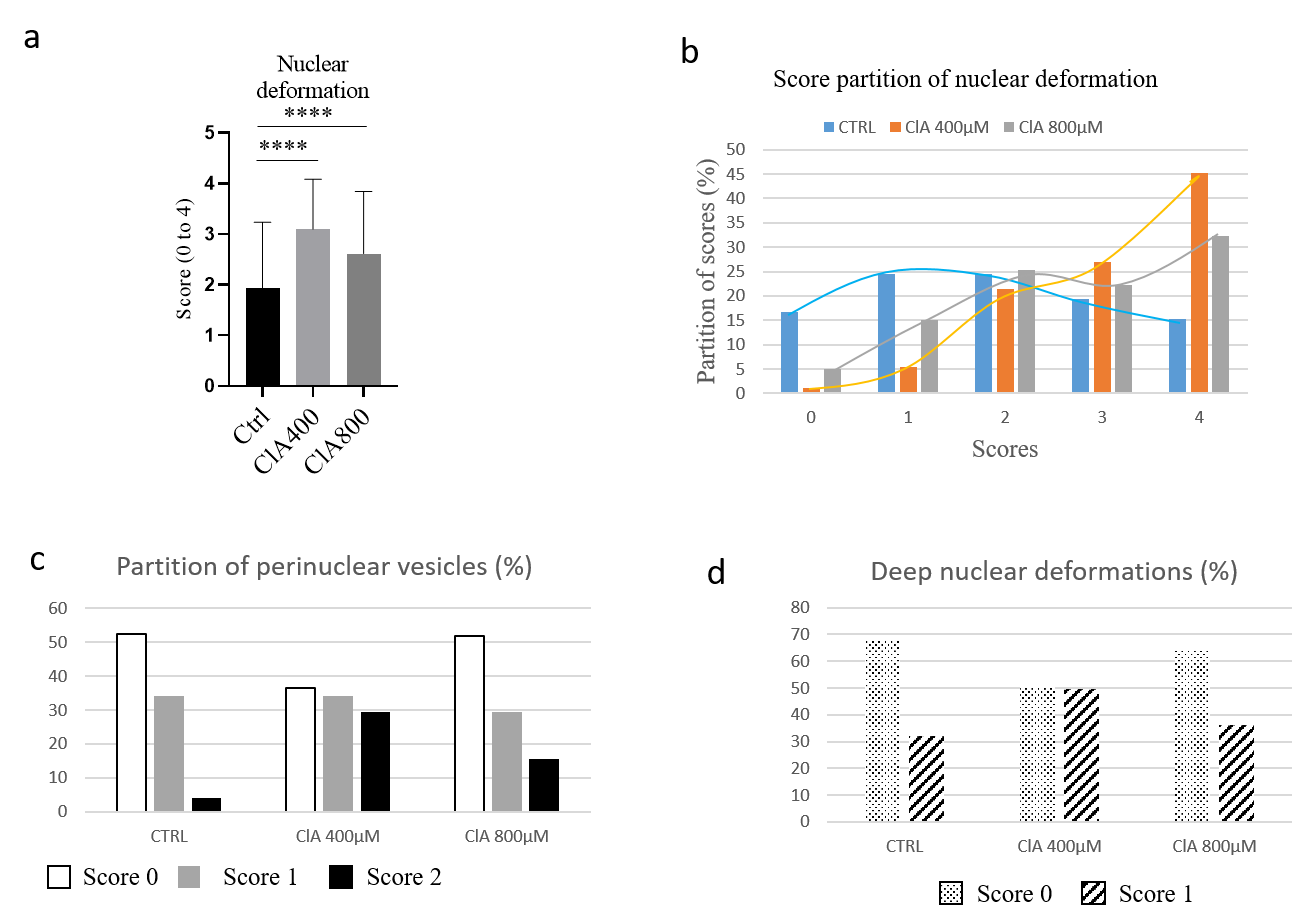
**

**Supplemental Fig. S7. Nuclear deformation in the granular keratinocytes of RHEs treated with chloro-amidine, an irreversible pan inhibitor of PADs.** RHEs were either untreated (Ctrl) or treated with 400 µM (ClA-400) or 800 µM (ClA-800) of the inhibitor. **(A)** Mean nuclear deformation scores of granular keratinocytes for Ctrl (216 nuclei), ClA-400 (93 nuclei) and ClA-800 (99 nuclei) RHEs (3 NHEK banks; ****, p < 0.0001). (**B)** Distribution of the nuclear deformation **s**cores for Ctrl (blue), ClA-400 (orange) and ClA-800 (grey) RHEs. (**C)** Distribution of the perinuclear vesicle scores (score 0, absence or very few and small vesicles; score 1, presence of some vesicles; score 2, presence of many and large vesicles). (**D)** Distribution of deep nuclear deformation scores (score 0, absence or score 1, presence of at least one deep nuclear deformation). n.s., non-significant. ****, *p* < 0.0001.

**
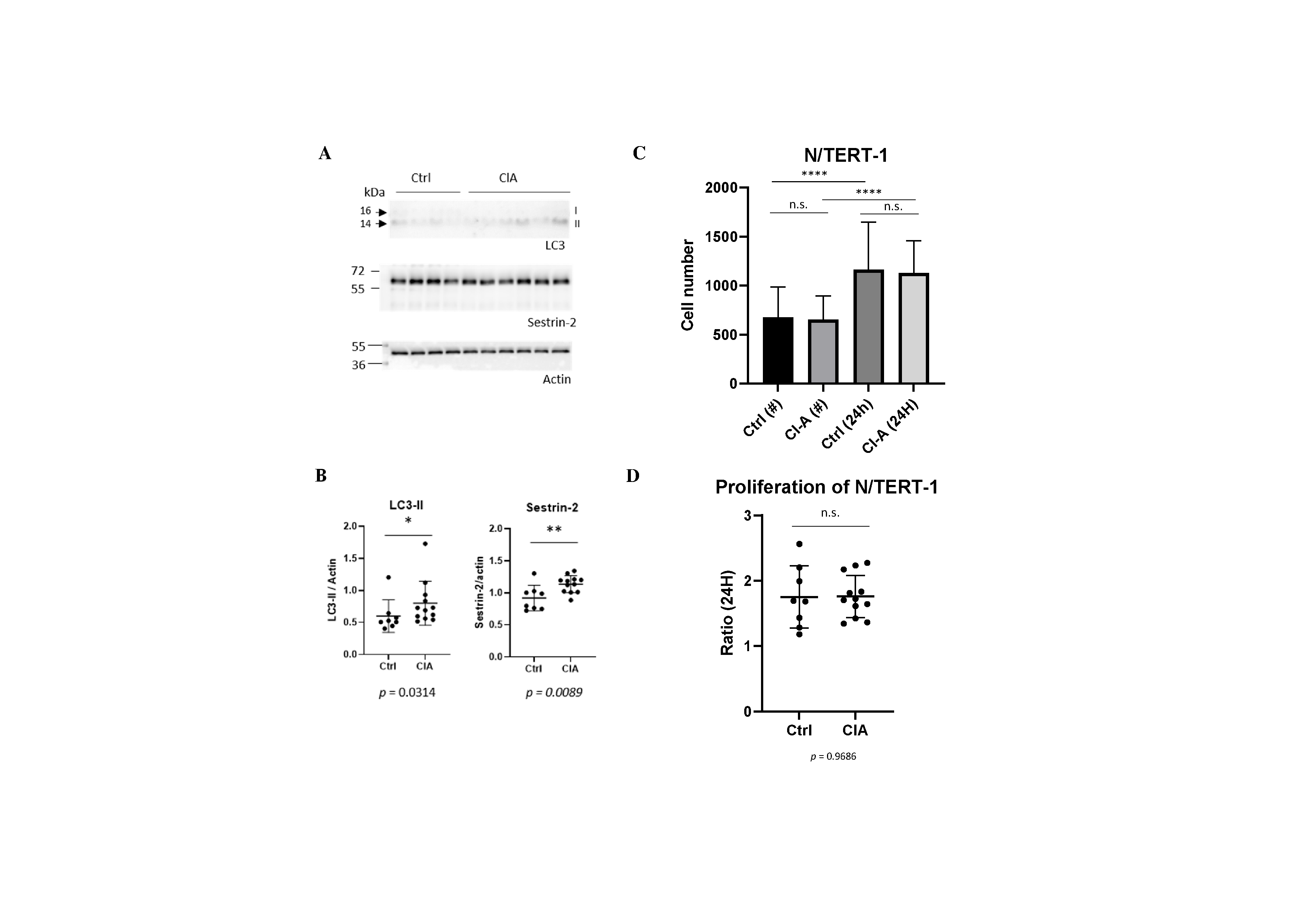
**

**Supplemental Fig. S8. No impact of chloro-amidine treatment on N/TERT-1 keratinocyte proliferation.** Proliferative human keratinocytes either untreated (Ctrl, n = 8) or treated with 100 µM chloro-amidine (ClA, n = 12) for 24 hours. **(A)** LC3B (top), sestrin-2 (middle) and actin (bottom) western blotting detections were performed to validate the treatment and (**B**) were quantified after normalization to actin. **(C)** N/TERT-1 cell number numerations before (#) and 24 hours after ClA treatments (****, *p* < 0.0001). **(D)** Individual N/TERT-1 proliferation ratios (after treatments/before treatments) in both conditions (*p* = 0.9686). Mean±SD were reported.

**Supplemental references**

s1. Dickson, M.A., Hahn, W.C., Ino, Y., Ronfard, V., Wu, J.Y., Weinberg, R.A., et al. Human keratinocytes that express hTERT and also bypass a p16(INK4a)-enforced mechanism that limits life span become immortal yet retain normal growth and differentiation characteristics. *Mol Cell Biol*. 20, 1436–1447 (2000).

s2. Serre G, Mils V, Haftek M, Vincent C, Croute F, A Réano A, et al. Identification of late differentiation antigens of human cornified epithelia, expressed in re-organized desmosomes and bound to cross-linked envelope. *J Invest Dermatol.* Dec;97(6):1061-72 (1991).

s3. Simon M, Sebbag M, Haftek M, Vincent C, Girbal-Neuhauser E, Rakotoarivony J, et al. Monoclonal antibodies to human epidermal filaggrin, some not recognizing profilaggrin. *J Invest Dermatol.* Sep;105(3):432-7 (1995).
