# Supplementary material for: Down-regulation of peptidylarginine deiminase type 1 in reconstructed human epidermis disturbs nucleophagy in the granular layer and affects barrier function
Source: Cell Death Discov. 2023 Jun 29;9:198. doi: 10.1038/s41420-023-01509-8 (PMC10310762; doi:10.1038/s41420-023-01509-8)
Supplement: Supplementary file 2 — Original Data File [file 41420_2023_1509_MOESM2_ESM.pptx]

## Slide 1
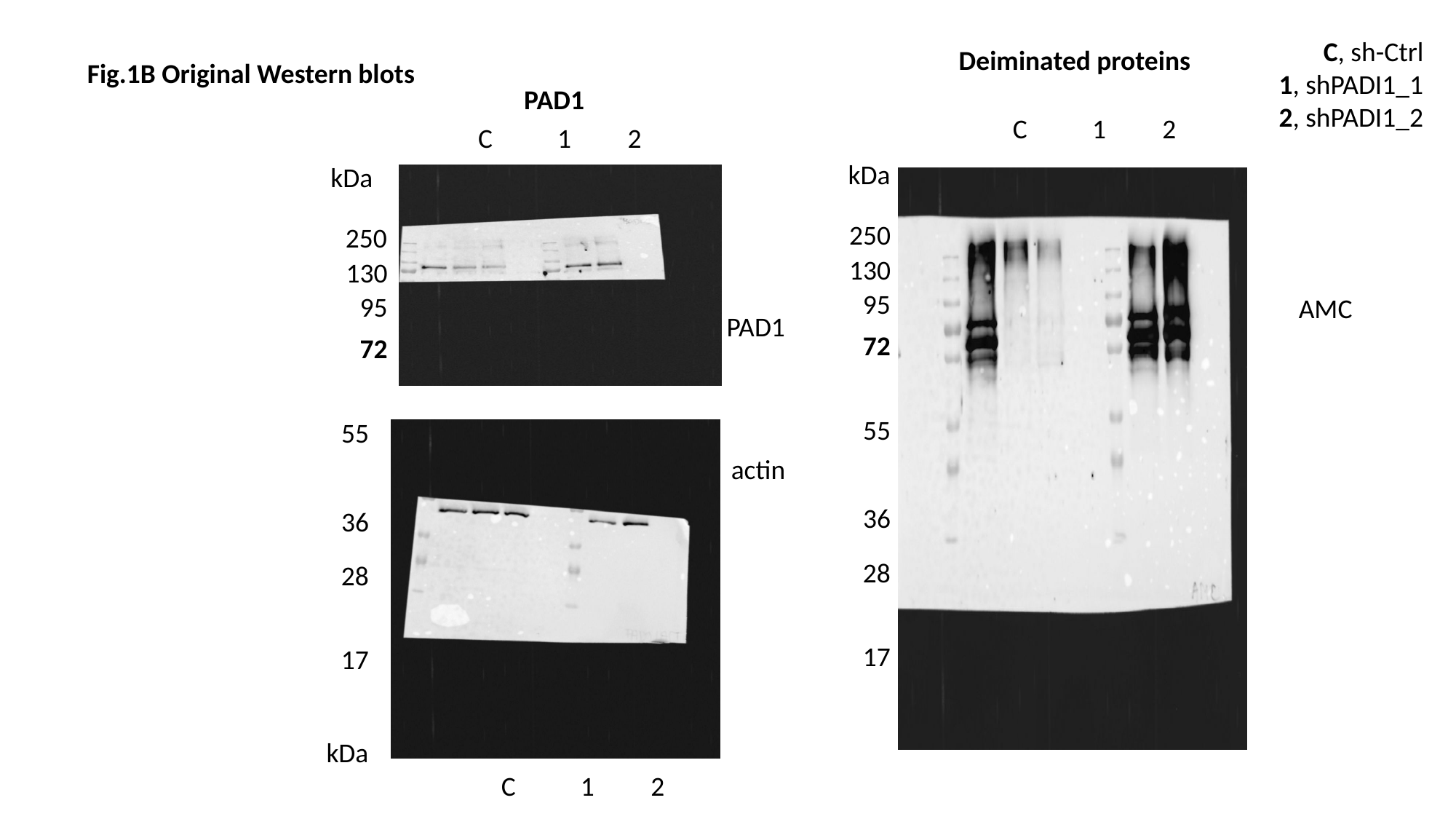

C, sh-Ctrl
1, shPADI1_1
2, shPADI1_2
Deiminated proteins
Fig.1B Original Western blots
PAD1
C
1
2
AMC
kDa
250
130
95
72
55
36
28
17
C
1
2
kDa
PAD1
250
130
95
72
55
actin
36
28
17
C
1
2
kDa

## Slide 2
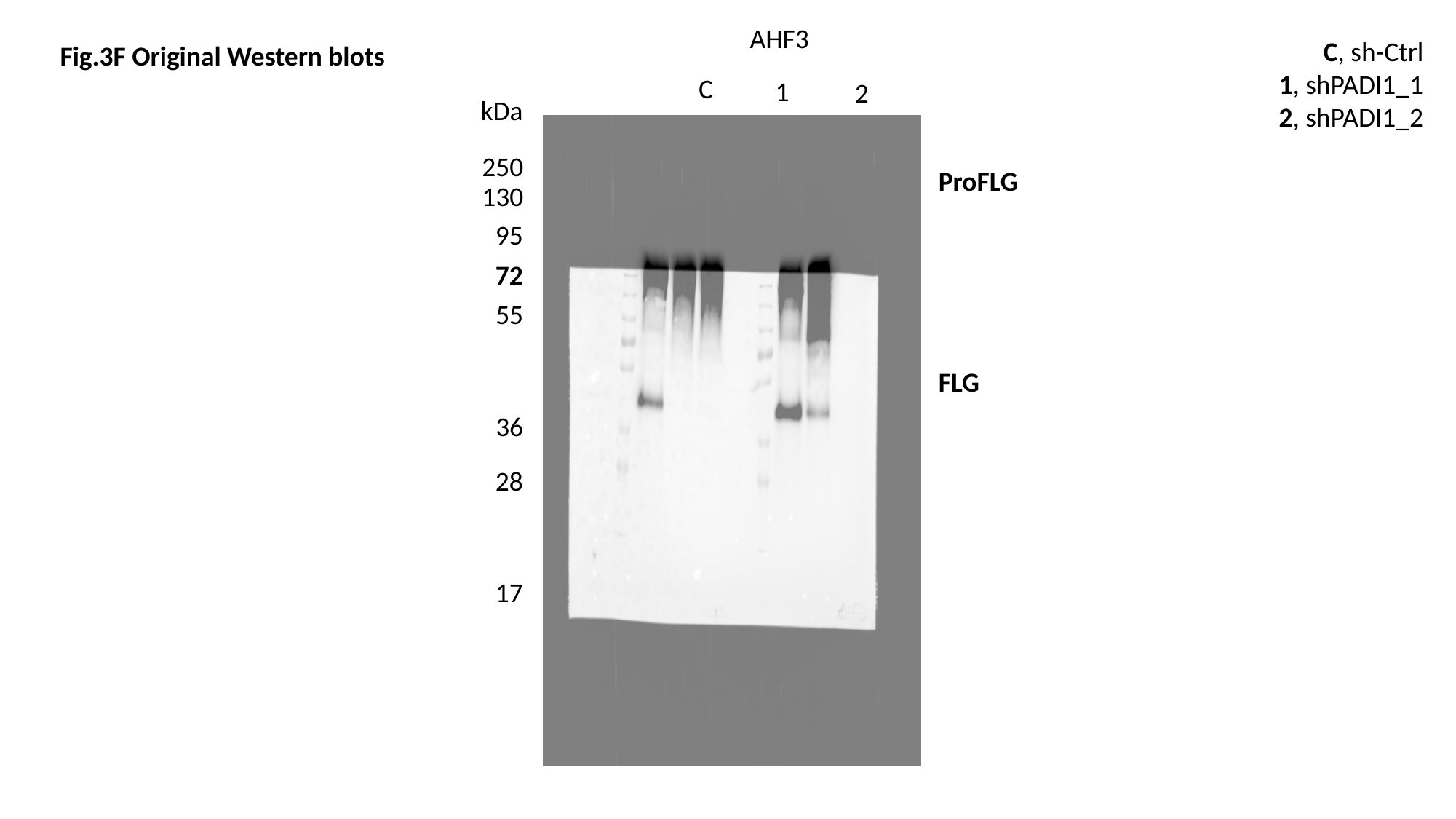

AHF3
C
1
2
kDa
250
ProFLG
130
95
72
55
FLG
36
28
17
C, sh-Ctrl
1, shPADI1_1
2, shPADI1_2
Fig.3F Original Western blots

## Slide 3
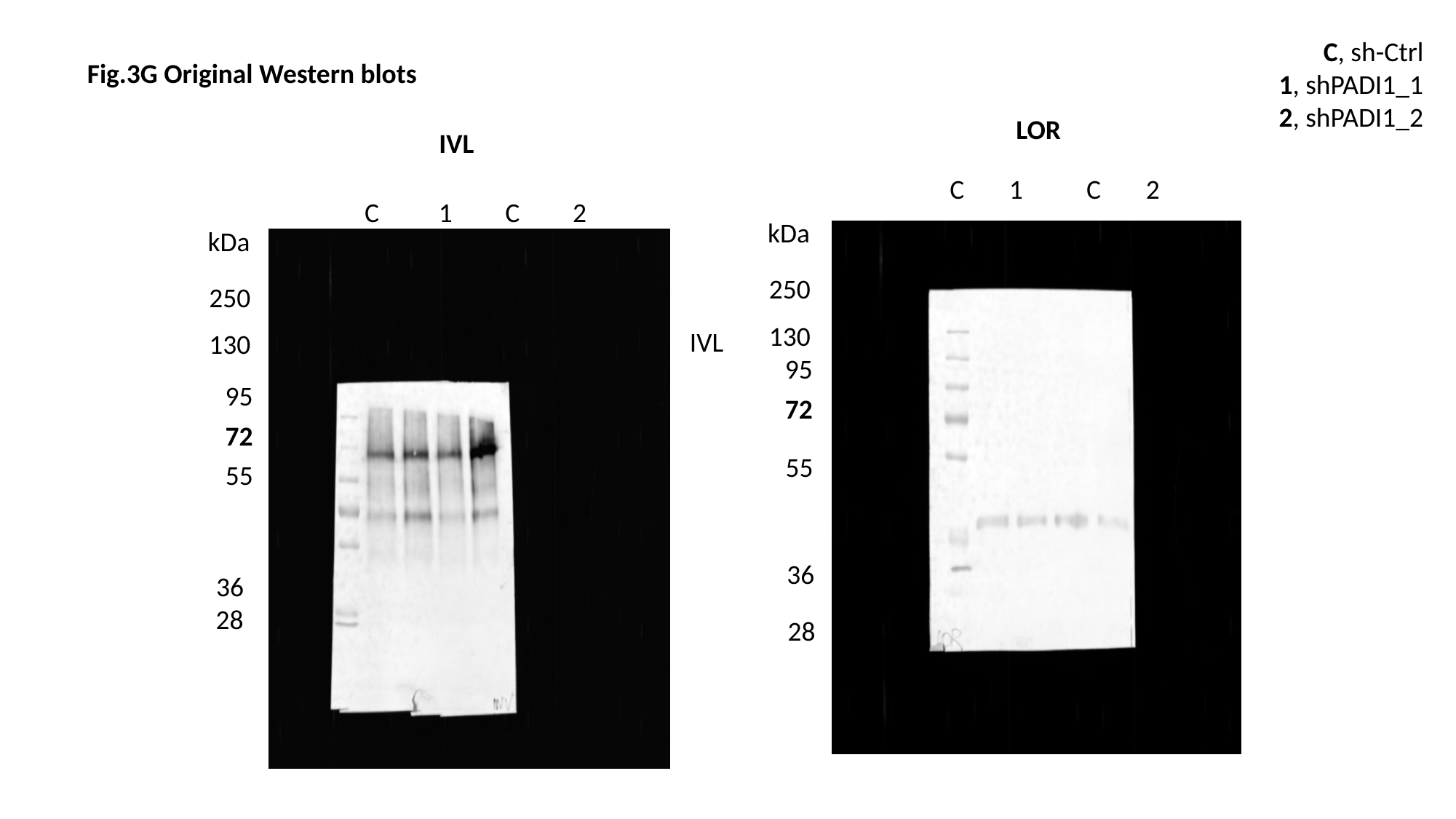

C, sh-Ctrl
1, shPADI1_1
2, shPADI1_2
Fig.3G Original Western blots
LOR
C
1
C
2
kDa
250
130
95
72
55
36
28
IVL
C
1
C
2
kDa
250
IVL
130
95
72
55
36
28

## Slide 4
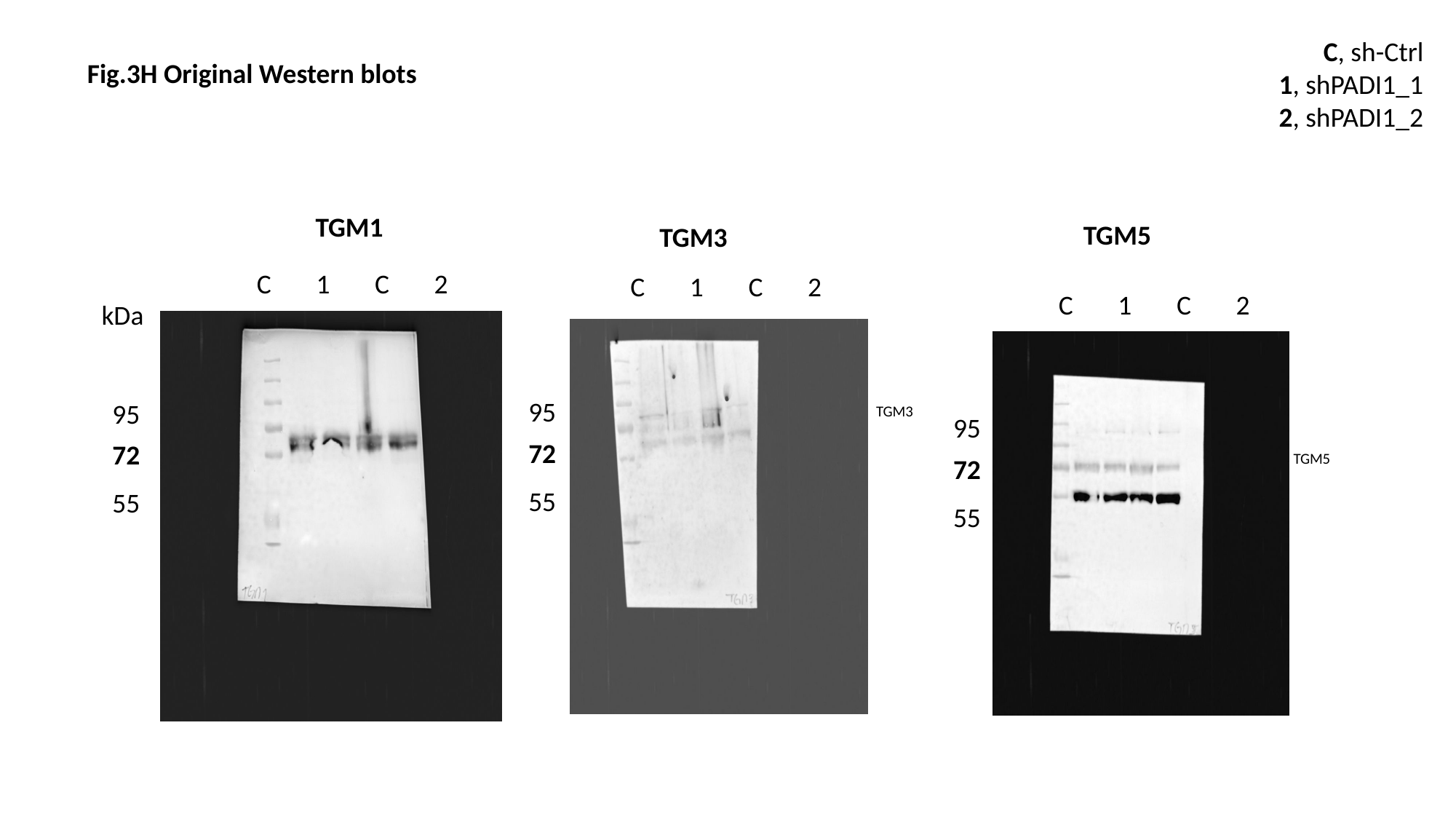

C, sh-Ctrl
1, shPADI1_1
2, shPADI1_2
Fig.3H Original Western blots
TGM1
C
1
C
2
kDa
95
72
55
TGM5
C
1
C
2
95
TGM5
72
55
TGM3
C
1
C
2
95
TGM3
72
55

## Slide 5
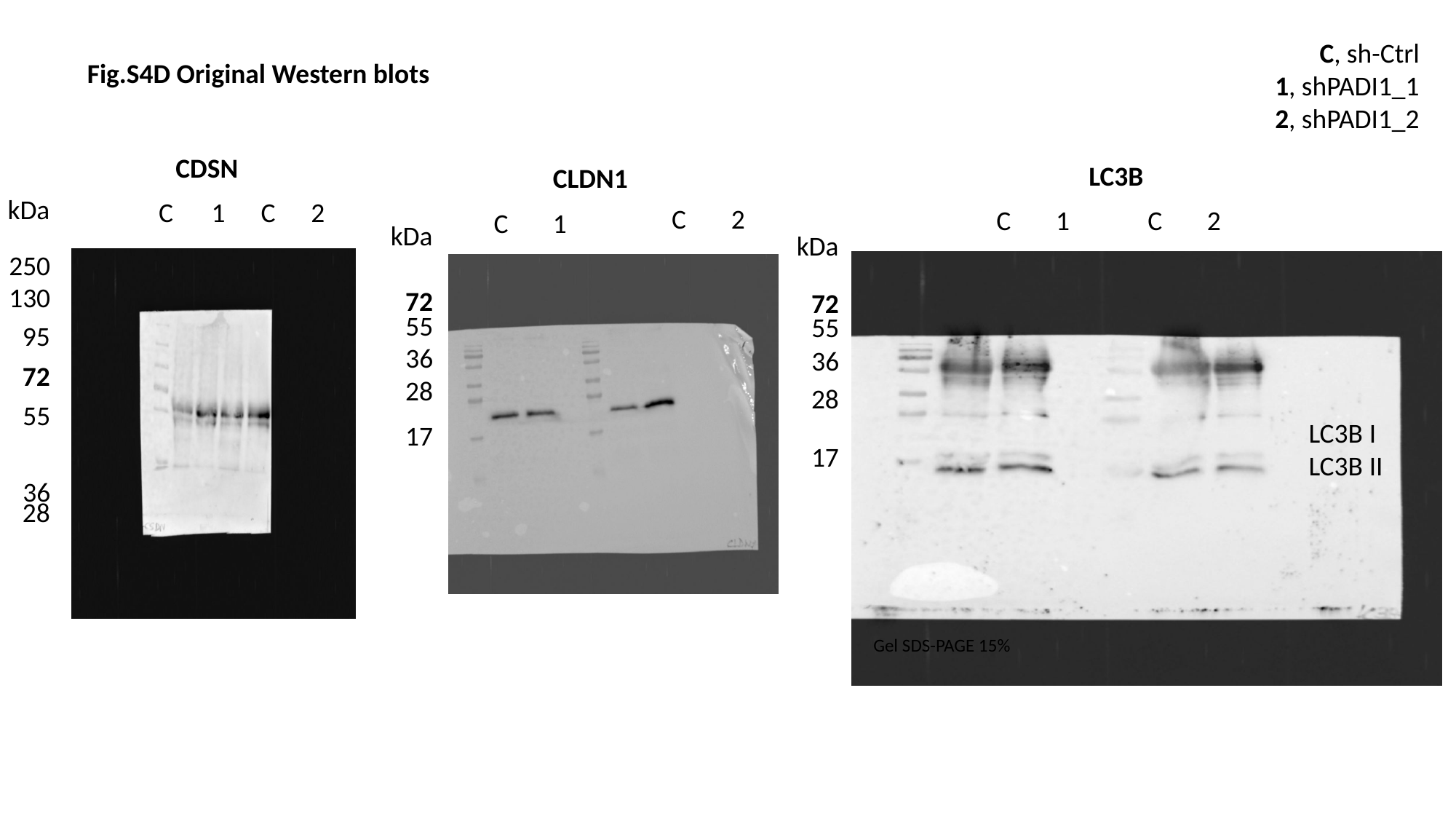

C, sh-Ctrl
1, shPADI1_1
2, shPADI1_2
Fig.S4D Original Western blots
CDSN
kDa
C
1
C
2
250
130
95
72
55
36
28
LC3B
C
1
C
2
kDa
LC3B I
LC3B II
72
55
36
28
17
Gel SDS-PAGE 15%
CLDN1
C
2
C
1
kDa
72
55
36
28
17

## Slide 6
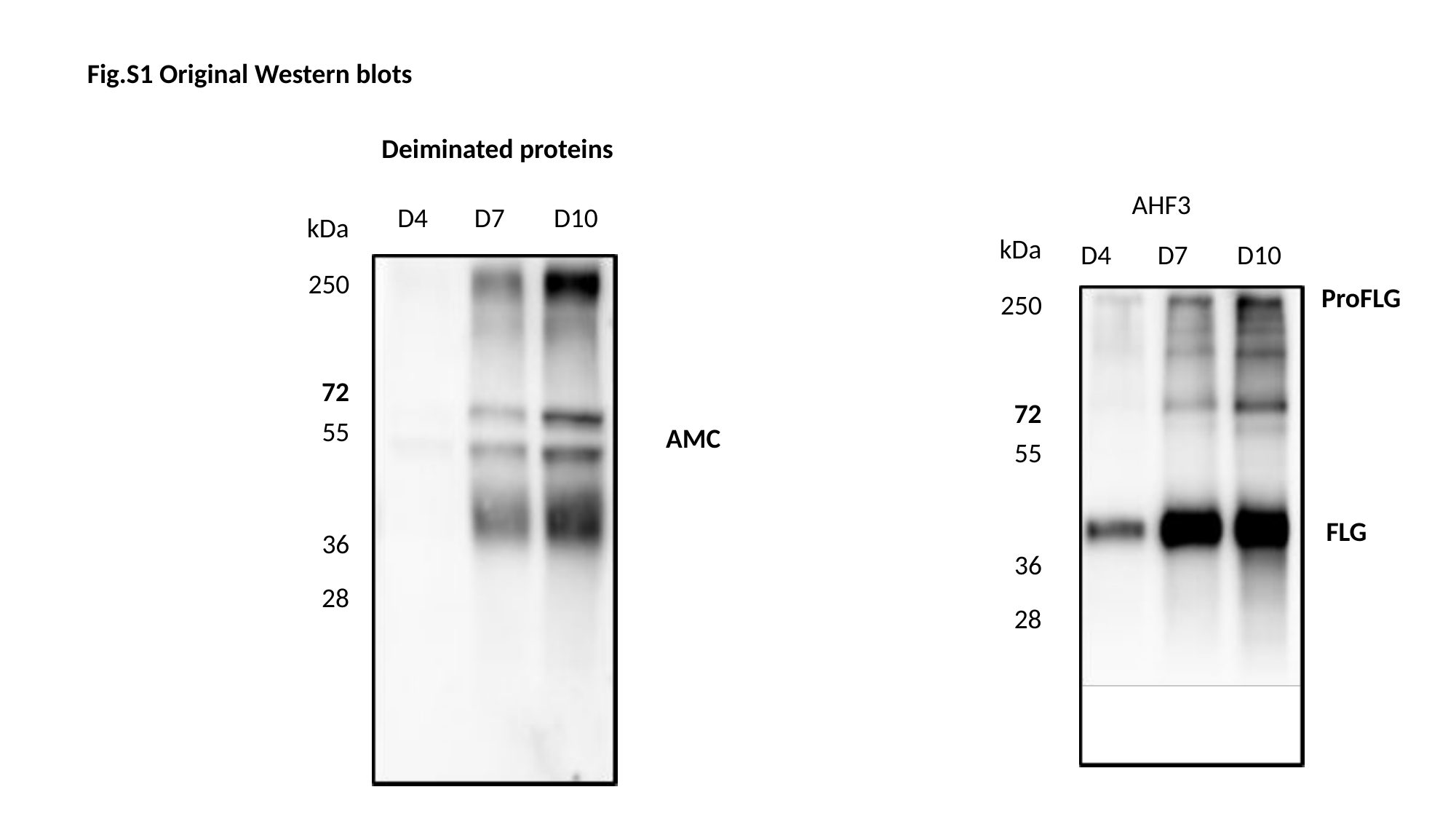

Fig.S1 Original Western blots
Deiminated proteins
D4
D7
D10
kDa
250
72
55
AMC
36
28
AHF3
kDa
D4
D7
D10
ProFLG
250
72
55
FLG
36
28

## Slide 7
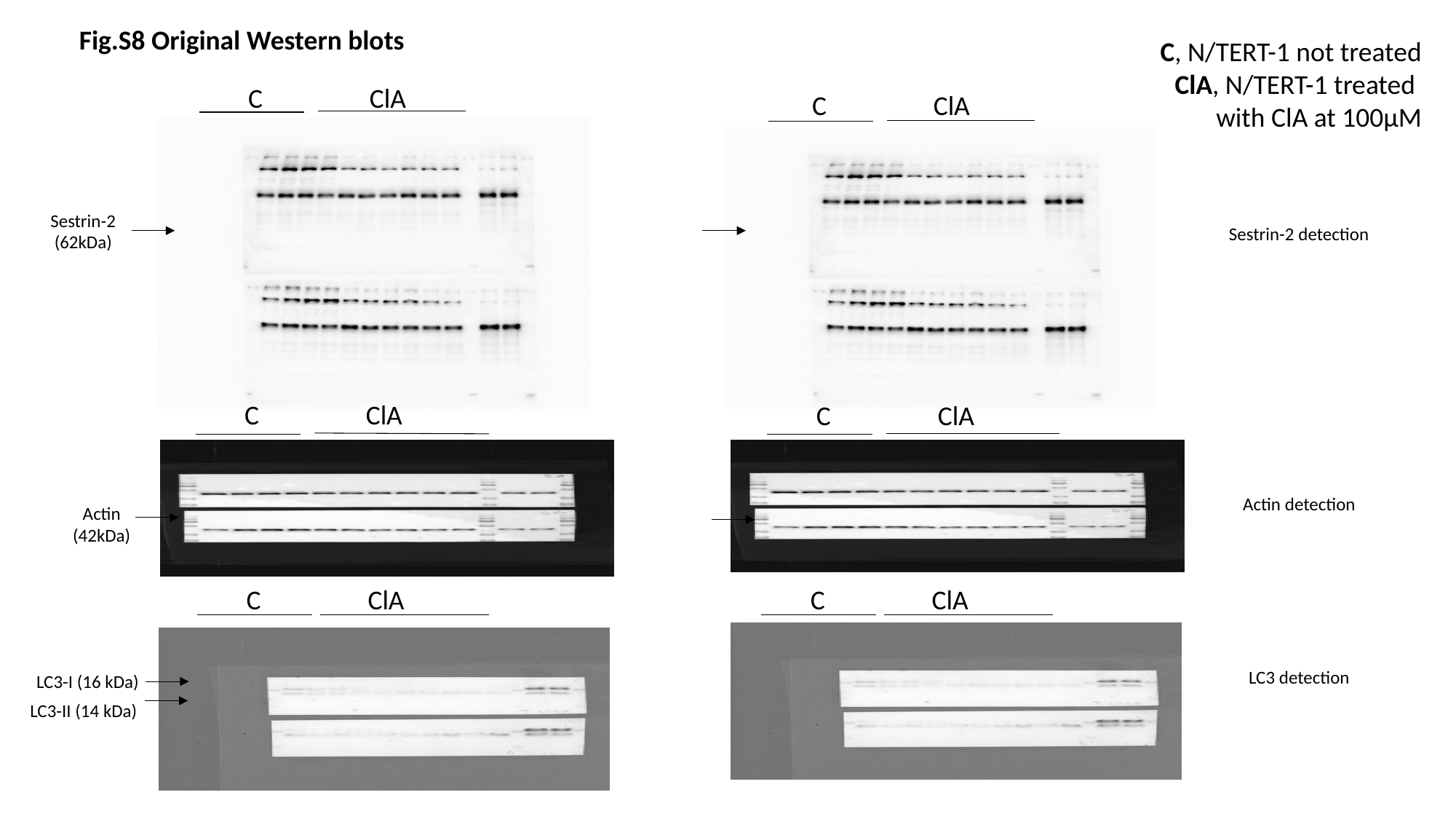

Fig.S8 Original Western blots
C, N/TERT-1 not treated
ClA, N/TERT-1 treated
with ClA at 100µM
C
ClA
C
ClA
Sestrin-2
(62kDa)
Sestrin-2 detection
C
ClA
C
ClA
Actin detection
Actin
(42kDa)
C
ClA
C
ClA
LC3 detection
LC3-I (16 kDa)
LC3-II (14 kDa)
